# Gene Expression Characteristics of Liver Tissue Reveal the Underlying Pathogenesis of Hepatocellular Carcinoma

**DOI:** 10.1155/2021/9458328

**Published:** 2021-10-04

**Authors:** Congfang Guo, Xiang Guo, Yudong Rong, Yirui Guo, Li Zhang

**Affiliations:** ^1^Physical Examination Center, Tianjin First Central Hospital, Tianjin 300192, China; ^2^College of Veterinary Medicine, Shanxi Agricultural University, Jinzhong 030801, China

## Abstract

**Background:**

Hepatocellular carcinoma (HCC) is high-mortality primary liver cancer and the most common malignant tumor in the world. This study is based on a hepatocellular carcinoma-related dysfunction module designed to explore the dysregulation of genes in liver cancer tissue.

**Methods:**

By downloading the relevant data on the GEO database, we performed a differential analysis of healthy liver tissue and liver cancer tissues as well as healthy liver tissue and hepatocellular carcinoma tissue and then obtained two sets of differential genes and combined them. We performed a cointerpretation analysis of these differential genes and constructed related functional disorder modules. A hypergeometric test was performed to calculate the potential regulatory effects of multiple factors on the module, and a series of ncRNA and TF regulators were identified. We obtained a total of 4479 differentially expressed genes in hepatocellular carcinoma, and these genes were clustered into ten hepatocellular carcinoma-related functional interpretation disorder modules.

**Results:**

Enrichment analysis revealed that these modular genes are mainly involved in signal transduction including cell cycle, TGF-beta signal transduction, and p53 signal transduction. Depending on the predictive analysis of multidimensional regulators, 323 ncRNAs and 52 TF-mediated hepatocellular carcinoma-related dysregulation modules were found to regulate disease progression.

**Conclusions:**

Based on a series of investigations, it was found that miR-30b-5p may participate in the peroxisome signal transduction by downregulating ABCD3-mediated module 1, thereby promoting the development and progression of hepatocellular carcinoma. Our research results not only provide a theoretical basis for biologists to study hepatocellular carcinoma further but also offer new methods and new ideas for the personalized care and treatment of hepatocellular carcinoma.

## 1. Introduction

Hepatocellular carcinoma (HCC) is a deadly human malignancy and one of the most common causes of cancer-related deaths worldwide. Hepatocellular carcinoma is a late stage disease associated with major vascular invasion, with a poor prognosis and low survival rate [1–3]. There are four main growth modes: trabecular type, solid type, pseudo/acinar type, and large and small beam type [4]. Hepatocellular carcinoma (HCC) is not only a heterogeneous malignancy but also a potentially fatal complication of chronic liver disease with limited treatment. Liver transplantation is the preferred treatment [5, 6]. The most common sites of extrahepatic metastases include the lung, regional lymph nodes, adrenal glands, and bone [7]. The limited treatment options for patients with advanced HCC have become a significant problem [8]. Most biologists have been involved in the pathological study of hepatocellular carcinoma. For example, Xu et al. observed that the genetic variation of the hepatitis B virus X gene (HBx) is associated with progression of hepatocellular carcinoma (HCC) and identified a novel HBx genotype that may result in the loss of proliferation-promoting function [9]. Epithelial-mesenchymal transition (EMT) is also associated with progression of hepatocellular carcinoma, which occurs during HCC progression [10]. According to previous reports, we found that regulatory T cells (Tregs) are an essential component of immune cell infiltration in the tumor microenvironment, which is closely related to the progression and metastasis of hepatocellular carcinoma [11]. Long noncoding RNAs (IncRNAs) have essential functions in the pathogenesis of hepatocellular carcinoma, including tumor growth, proliferation, metastasis, invasion, and recurrence [12]. The occurrence and development of diseases are often related to the interpretation of genes. The interpretation of SNHG16 was significantly increased in HCC tissues and cell lines, and its overexpression was inseparable from the invasive clinicopathological features and poor prognosis of HCC patients [13]. Zhong et al. [14] found that upregulated interpretation of miR-21-5p may be a functional regulator of metabolism or apoptosis in HCC and is also a novel tumor marker for the early diagnosis of HCC. Besides, studies have shown that CircPTGR1 is a circRNA with three isoforms. CircPTGR1 is upregulated in serum exosomes of HCC patients and is associated with clinical stage and prognosis [15]. In a report by the scholar (Wang J), we found that pescadillo ribosomal biosynthesis factor 1 (PES1) is generally upregulated in HCC tissues and cells, may promote proliferation and tumorigenicity, and represents a novel prognostic marker for HCC overall survival [16]. Signal transductions also have essential regulatory functions in disease progression. Cancer stem cells (CSCs) have been identified as a small fraction of cancer cells with high self-renewal, differentiation, and tumorigenic capacity [17]. CD44v is the major isoform expressed on CSCs of various tumors and has been extensively studied. The data suggest that CD44s may be involved in the maintenance of CSC in HCC cell lines via the NOTCH3 signal transduction [18].

According to a study by a scholar (Guerrero M), we found that the mTOR pathway is overexpressed in patients with multinodular HCC and is associated with an increased tumor recurrence rate after LT (liver transplantation) [19]. According to the above-mentioned partial pathogenesis of hepatocellular carcinoma, scientists and medical personnel from all over the world are actively exploring corresponding treatments and personalized care. In the treatment of hepatocellular carcinoma, scientists such as Xu et al. pointed out that FHIT and p16 as tumor suppressor genes can effectively inhibit the proliferation of HCC, which can be used as a new indicator for clinical detection, providing an original method for clinical diagnosis [20]. The circular RNA (circRNA) hsa_circ_0079299 is considered to be overexpressed in hepatocellular carcinoma, inhibiting tumor growth in vitro and in vivo and delaying cell cycle progression. Its tumor-suppressive effect in HCC provides a new understanding of circRNA in cancer [21]. The latest advances in nanotechnology are milestones in the study of treatments for hepatocellular carcinoma. According to the study of Chi et al., the integration of imaging and treatment of arsenite-containing magnetic mesoporous silica nanoparticles has great promise for the treatment of hepatocellular carcinoma [22]. Recent studies have found that rosiglitazone metformin adduct (RZM) increases the antiproliferative effect of metformin (Met) in HCC by upregulating p21 interpretation in an AMPK-dependent manner. The results indicate that RZM may be an adjuvant for the effective treatment of HCC [23]. Besides, raloxifene inhibits the growth of hepatocellular carcinoma in vitro and in vivo, suggesting another potential strategy for HCC prevention and treatment [24]. Previous clinical studies have shown that low serum cholesterol can predict adverse outcomes in HCC patients. Cholesterol inhibits the progression of HCC by inhibiting SCAP-mediated new fatty acid synthesis [25]. In the personalized care of hepatocellular carcinoma, we should consider the burden and extent of HCC. We also discuss the patient's performance status, potential liver function, extrahepatic disease and comorbidity, and the stage of HCC at diagnosis and provide multiple treatment options and determine the best treatment option [26]. Barcelona's advanced clinical liver cancer (BCLC C) is a type of hepatocellular carcinoma staging, and the best-recommended treatment is to use sorafenib alone [27].

Percutaneous ablation and hepatectomy are recommended first-line treatment options depending on the extent of liver resection and liver function. Laparoscopic surgery (resection or ablation) is an excellent surgical procedure when the tumor is located on the surface of the liver and close to the extrahepatic organ [28]. For HCC sample studies, CYP3A4 immune response was observed in peripheral hepatocytes, and CYP3A7 immunostaining was found in normal hepatocytes. CYP3A4 metabolizes sorafenib but not by CYP3A7. Overexpression of CYP3A4 may lead to increased drug degradation, which in turn leads to clinical ineffectiveness [29]. These results suggest that positive CYP3A4 in HCC liver samples may indicate a rare response to sorafenib, suggesting the need for individualized treatment of HCC [30]. Based on the gene interpretation characteristics and functional pathways of liver tissue, we propose a comprehensive approach. Based on coexpression analysis and enrichment analysis of functional pathways, we focused on a series of core ncRNA and transcription factor regulators and revealed the underlying pathogenesis of hepatocellular carcinoma to guide personalized care and treatment.

## 2. Materials and Methods

### 2.1. Data Resource

Data interpretation profiles for hepatocellular carcinoma were obtained from GSE67764 in the NCBI Gene interpretation Omnibus database [31]. In the dataset, a gene assay to screen the expression of total RNA in liver tissues, including 3 normal liver tissues, 3 HCC paracancer specimens, and 3 HCC samples with HBV-induced hepatitis. The normal liver samples were obtained from normal hepatic tissue in three hepatic hemangioma surgical operative patients, and three matched pairs of HCC and its paracancer tissues were, respectively, obtained from three HCC surgical operative patients without any chemotherapy and radiotherapy. The dataset contains normal and disease samples from different tissues of hepatocellular carcinoma [32]. There are 3,600 related genes in cancer tissues and 1224 associated genes in adjacent tissues. To understand the genetic characteristics of liver tissue, we integrated the genes of these two different tissues to obtain a final set of differential genes.

### 2.2. Coexpression Analysis Identifies Relevant Functional Modules

To explore the synergistic interpretation of the dysregulated genes, we constructed a gene interpretation profile matrix for cointerpretation network analysis (WGCNA) of these genes [33]. WGCNA is a system biology method used to describe patterns of gene association between different samples. It can be used not only to identify synergistic changes in gene sets with high intrinsic but also to communicate the link between gene interpretation behavior and sample phenotype. We used the correlation coefficient weighting value; that is, the gene correlation coefficient is taken to the *N*th power, and the correlation coefficient (Pearson's coefficient) between any two genes is calculated. Since the nodes in the network are subject to scale-free systems, their characteristics are consistent with the interpretation relationship between genes, so the algorithm is more biologically significant than other algorithms. Correlation coefficients between genes construct a hierarchical clustering tree, and different branches of the clustering tree represent different gene modules; that is, different colors represent different modules.

### 2.3. Functional and Pathway Enrichment Analysis to Identify Dysfunction Modules

The exploration of the functions of genes and the exploration of participating signal transductions are often effective means of studying the molecular mechanisms of disease. The function and pathway involved in the module gene can characterize the dysfunction mechanism of the module during the disease process. For each functional module gene of hepatocellular carcinoma, using the Cluster profile package [34], we performed Go function (*p* value cutoff = 0.05, qvalueCutoff = 0.05) and KEGG Pathway (*p* value cutoff = 0.05, qvalueCutoff = 0.05) enrichment analysis. We screened the functions and pathways reported to be associated with the progression of hepatocellular carcinoma and mapped the bubbles.

### 2.4. Identification of Transcription Factors and ncRNA Regulation of Modules

Data on transcriptional and posttranscriptional target regulation relationships are included in the TRRUST v2.0 database and the RAID v2.0 database [35, 36]. TRRUST v2 is now the most comprehensive database based on manual curation for both human and mouse TF–target interactions. RAID v2.0 is aimed at providing a comprehensive and reliably assessed collection of RNA-associated interactions across organisms. Among them, we downloaded and used all human transcription factor target data in the TRRUST v2 database, involving 52 transcription factors and 52 TF-Module interaction pairs. In RAID 2.0, we set the interaction score > 0.5 and screened 323 ncRNA-Module interaction pairs on humans, involving 356 ncRNAs. These regulators often mediate disease development. To explore the driving forces of cointerpretation modules for hepatocellular carcinoma-associated genes, we performed a pivotal analysis based on these interaction data. Pivot analysis refers to the search for a driver with at least two pairs of modules in the target pair, and we calculate the significance of the interaction between the driver and the module based on the hypergeometric test. The ncRNAhe1TF with *p* value < 0.01 is the pivot of the significant regulatory module. We performed a statistical analysis of the pivot, and the pivotal function of the dysfunction module was identified as the core pivot. The ncRNA and TF-based target data were predicted as background sets, and the crucial regulator of the regulatory dysfunction module was obtained.

## 3. Results

### 3.1. Determining the Time Series Expression of Hepatocellular Carcinoma

Gene interpretation disorders have essential functions in the disease process. To explore the genetic dysregulation of hepatocellular carcinoma, we screened differentially the interpretation profile of hepatocellular carcinoma. We obtained two sets of differences and combined the two groups of differential genes, with a total of 4479 (Table S1). Also, these differentially expressed genes may have a direct/long association with hepatocellular carcinoma, and they may have essential regulatory functions in the development of the disease.

### 3.2. Identify Functional Hepatocellular Carcinoma Staging-Related Modules

**Figure 1:**
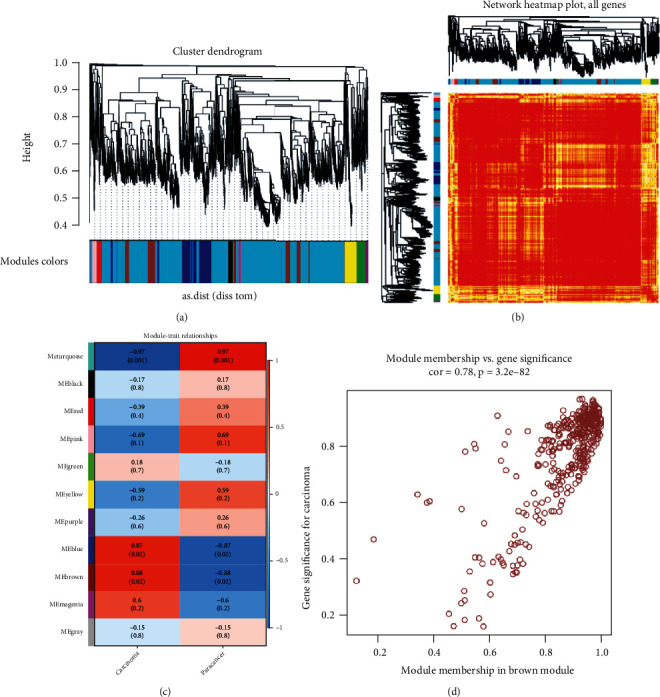
(a) Cointerpretation analysis clustered hepatocellular carcinoma-related differential genes into ten modules. The ten colors represent ten modules. (b) Cluster gene interpretation heat map of the module gene in the sample. (c, d) Each row represents a module, and each column represents a phenotype. The corresponding correlation coefficient maps the color of each cell. Values range from -1 to 1; the color changes from blue to white. The transition to red is a significant positive and negative correlation of the module. The darker the color, the more prominent it is in the module.

We constructed interpretation profiles in patient samples for 4479 differentially expressed genes in hepatocellular carcinoma. Based on the WGCNA network for cointerpretation analysis of differentially expressed genes, ten cointerpretation modules were obtained, and these module genes showed significant clustering in the samples (Figures 1(a) and 1(b)). According to the association phenotypic analysis, we found that the first module was positively correlated with the potential pathogenesis triggered by hepatocellular carcinoma (Figures 1(c) and 1(d)). Through the introduction of differential genes into the module gene, we obtained a hub gene with strong regulation of hepatocellular carcinoma in each module and considered that these genes have essential regulatory functions in the pathogenesis of hepatocellular carcinoma. Functional modules may be involved in different functions and pathways and represent different regulatory mechanisms that mediate the development and progression of hepatocellular carcinoma.

### 3.3. Functional and Pathway Enrichment Analysis to Identify Dysfunction Modules

**Figure 2:**
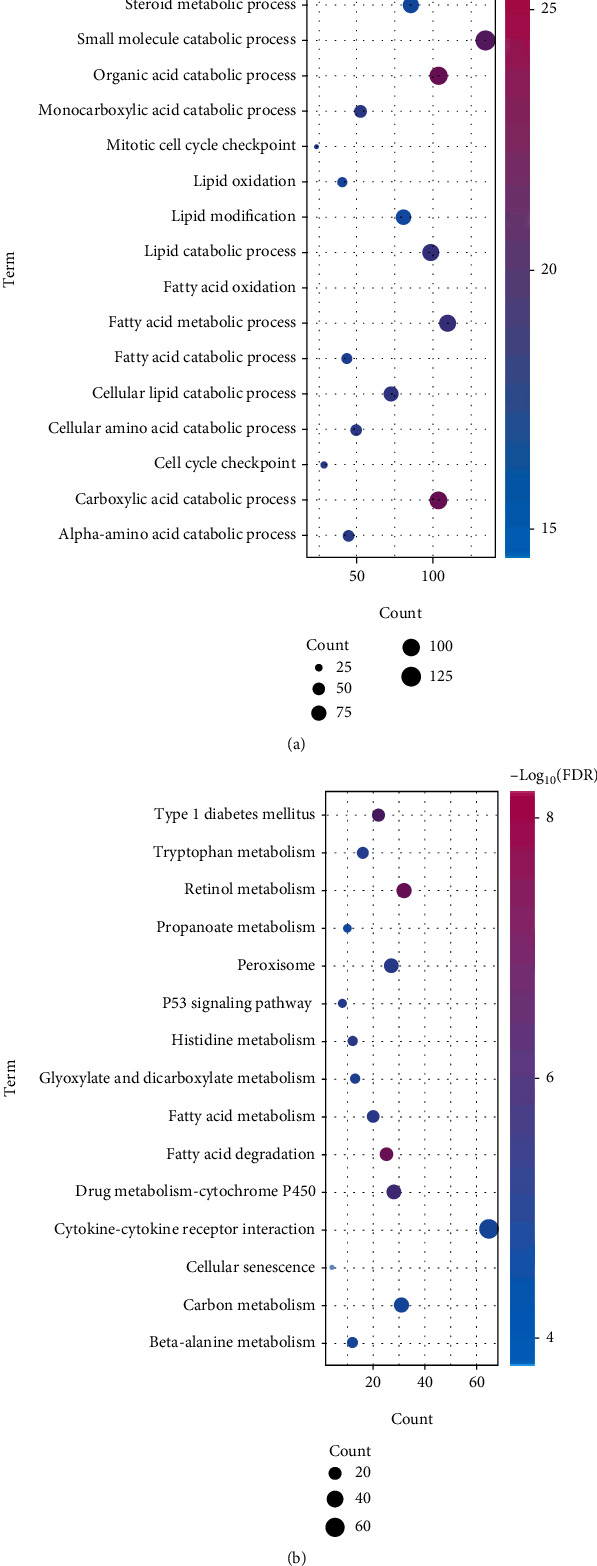
Modules involved in the function and pathway of the gene to identify hepatocellular carcinoma dysfunction modules. (a) Module gene GO function enrichment analysis. The deeper the color, the stronger the enrichment. The larger the circle, the more significant the proportion of the gene in the module that accounts for the GO function. (b) Module gene KEGG pathway enrichment analysis. The deeper the color, the stronger the enrichment. The larger the circle, the more significant the proportion of the gene in the KEGG pathway entry.

Studying the functions and pathways involved in genes is an essential means of identifying their pathogenesis. To analyze the possible dysfunction of modular gene imbalance, we performed separate enrichment analysis of functions and pathways for each module. The results showed that most of the functional modules were enriched in hepatocellular carcinoma-related functions and pathways. We performed GO function and KEGG pathway enrichment analysis on the obtained ten functional modules and received a total of 36,231 functions and 1247 KEGG pathway enrichment results. It includes 5569 molecular functions (MF), 3281 cell components (CC), and 27,381 biological processes (BP) involved in the gene (Table S2, Figures 2(a) and 2(b)). Signal transductions include cell cycle, fatty acid metabolism, TGF-beta signal transduction, and p53 signal transduction. It may be identified as a core signal transduction involved in its potential disorder mechanism. We found that these signal transductions may be closely related to the genetic profile of liver tissue in the pathogenesis of hepatocellular carcinoma.

### 3.4. Identification of Critical Regulators Driving the Development in Hepatocellular Carcinoma

**Figure 3:**
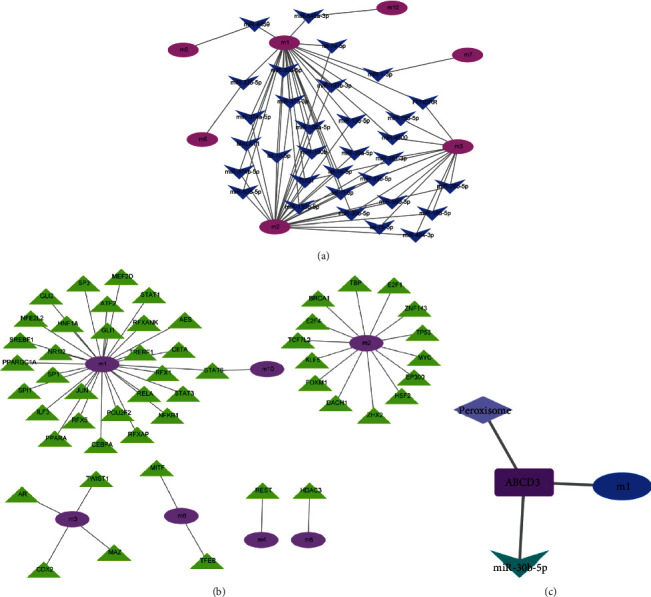
(a) Modulation of noncoding RNA to dysfunction modules. The color from pink to blue represents the connectivity of the nodes from large to small, that is, the more complicated the relationship with the regulation. (b) Regulatory effects of transcription factors on dysfunction modules. The color from purple to green represents the connectivity of the nodes from large to small and the more complicated the relationship with regulation. (c) Essential genes of hepatocellular carcinoma may participate in related signal transductions through target gene-mediated modules to promote the occurrence and development of diseases.

Hepatocellular carcinoma is a complex disease with multiple factors and multiple cascades, and a variety of factors naturally regulates the functional disorder module of hepatocellular carcinoma. ncRNA is considered to be an essential regulator. Scientific prediction of ncRNAs that regulate dysfunction module genes facilitates our in-depth exploration of the transcriptional regulatory mechanisms of hepatocellular carcinoma. We based on cpRNA-based pivot analysis to explore the ncRNA regulators that cause dysfunction of the module. The predicted results (Table S3, Figure 3(a)) show that 323 ncRNAs have significant regulatory effects on the module, involving 356 ncRNA-Module target pairs. These ncRNAs affect the development of hepatocellular carcinoma to varying degrees. Besides, statistical analysis of the results revealed that miR-30b-5p and TUG1 have significant regulatory relationships with the three functional disorder modules and have essential functions in the dysfunction of the module. ncRNA (FENDRR, let-7b-5p, and let-7c-5p) exhibits a significant regulatory effect on dysfunction modules and also has critical functions in the progression of hepatocellular carcinoma. The occurrence and development of hepatocellular carcinoma are also inextricably linked to the dysregulation of transcription factors, which is also reflected in the regulation of transcription factors to dysfunction modules. Therefore, we perform a pivot analysis of the module based on the regulatory relationship of the transcription factor to the gene. The results showed that (Table S4, Figure 3(b)) a total of 52 transcription factors have significant transcriptional regulation on hepatocellular carcinoma dysfunction modules, involving 52 TF-Module regulatory pairs. For transcription factor regulatory pairs, we performed statistical analysis. We found that STAT6 mediates two modules, while TFs such as JUN, AES, and AR mediate a module that affects the development and progression of hepatocellular carcinoma. We also found that miR-30b-5p, an essential gene affecting hepatocellular carcinoma, may contribute to the development and progression of hepatocellular carcinoma by targeting ABCD3-mediated module 1 involvement in the peroxisome signal transduction (Figure 3(c)).

## 4. Discussion

Hepatocellular carcinoma (HCC) is one of the most common cancers in the world. This malignant tumor is associated with poor prognosis and high mortality, especially in the Chinese population [37, 38]. Liquid biopsy is noninvasive and allows repeated analyses to monitor tumor recurrence, metastasis, or treatment responses in real time. With the advanced development of new molecular techniques, HCC circulating tumor cells (CTCs) and circulating tumor DNA (ctDNA) detection have achieved interesting and encouraging results. Based on the gene interpretation characteristics of liver tissue, we reveal that the pathogenesis of hepatocellular carcinoma guides personalized care and treatment and proposes a comprehensive method. Four thousand four hundred seventy-nine potential pathogenic genes (including MYH2, FGF21, and CYP26A1) for hepatocellular carcinoma were integrated, and cointerpretation analysis was performed with a correlated phenotype. We identified ten functional modules with synergistic interpretation and obtained the corresponding hub genes (CDCA5, TPTE2) for each module. Comprehensive studies have shown significant changes in eight protein spots in patients with hepatocellular carcinoma. Four proteins were successfully identified, including MYH2 protein, mitochondrial ATP synthase, sulfated glycoprotein-2 (SGP-2), and glial fibrillary acidic protein (GFAP). These results indicate that MYH2 protein, mitochondrial ATP synthase, SGP-2, and GFAP may be potential molecular biomarkers of hepatocellular carcinoma [39]. Also, fibroblast growth factor 21 (FGF21) has essential functions in liver metabolism and is also a potential marker for nonalcoholic fatty liver disease (NAFL). High fat, the high sucrose-induced fat-induced elevation of FGF21, may have essential functions in inhibiting hepatic pathology from NAFL to HCC [40]. During the development of HCC, decreased levels of FGF21 protein are associated with cancerous hyperproliferation and abnormal p53 and TGF-*β*/Smad signaling [41]. Data analysis of Lepri SR revealed that HepG2/C3A cells were exposed to genistein (5 and 50 *μ*M).

After 12 hours, CYP1A1 and CYP1B1 were upregulated, and CYP2D6 and CYP26A1 and CYP26B1 mRNA levels were downregulated [42]. Studies have found that increased CDCA5 interpretation is not only associated with increased HCC tumor diameter and microvascular invasion but may also be associated with hepatocellular carcinoma [43]. Further studies have shown that depletion of CDCA5 can reduce the levels of ERK1/2 and AKT phosphorylation *in vitro* and *in vivo* [44]. In conclusion, CDCA 5 may be a biomarker and therapeutic target for liver cancer prognosis [45]. Based on the SNP analysis of Clifford RJ, we identified two variants while recognizing that their allele frequencies differ essentially between HCC and cirrhosis (LC). One of them is located in the PTEN homolog TPTE2 [46]. Given functional enrichment results, we found that the signal transduction that modules tend to participate includes cell cycle, fatty acid metabolism, TGF-beta signal transduction, and p53 signal transduction. According to related studies, saffron inhibits the growth of QGY-7703 cells and stops the cell cycle in the G0/G1 phase and induces cell apoptosis [47]. A-Raf and fatty acid 2-hydroxylase (FA2H) are expressed in the early stage of liver cancer, both during progression and in tumor stage. The results indicate that elevated interpretation of A-Raf and FA2H in hepatocellular carcinoma is associated with lipid metabolism disorders and cancer progression [48]. Bi et al. believe that miR-181a-5p is a negative regulator of Egr1 and can inhibit tumor proliferation in HCC by targeting the Egr1/TGF-*β*1/Smad pathway, which may be a potential therapeutic approach for HCC [49]. The ubiquitin-binding enzyme E2S (UBE2S) has important functions in the development of human cancer. Pan et al. demonstrated in vitro experiments that UBE2S overexpression promotes proliferation and migration of HCC cells. UBE2S knockdown inhibits HCC cell proliferation and migration by regulating the p53 signal transduction [50]. These functions and pathways involved in the modular gene produce a far-reaching network effect that comprehensively regulates the pathogenesis of hepatocellular carcinoma. We explored a range of drivers for these dysfunction modules involving ncRNA (miR-30b-5p, TUG1, FENDRR, let-7b-5p, and let-7c-5p) and transcription factors (STAT6, JUN, AES, and AR). In terms of the driving force of ncRNA, miR-30b-5p and TUG1 both regulate the three functional disorder modules. Based on clinical data, miR-30b-5p was found to be associated with a variety of clinicopathological features (survival time, tumor size, pathological stage, differentiation, and intrahepatic metastasis). It also has an inhibitory effect on cell proliferation and cell cycle of HCC cell lines [51]. Long-chain noncoding RNAs (lncRNAs) have been reported to have key regulatory functions in the development of various cancers. General studies have shown that long noncoding RNA TUG1 is essentially upregulated in HCC tissues and cell lines; knocking out TUG1 may inhibit cell proliferation, cell migration, cell invasion, and epithelial-mesenchymal transition (EMT) [52]. Also, TUG1 was confirmed to be a molecular sponge of miR-144. It was found that TUG1 interacts with miR-144 to promote proliferation and migration of HCC cells by activating the JAK2/STAT3 pathway *in vitro* [53]. The above results indicate that TUG 1 can be used as a new diagnostic biomarker and therapeutic target for HCC [54]. In the regulation of transcription factors, STAT6 essentially regulates two functional disorder modules, while TF such as AR and ATF2 has a driving effect on one module, which affects the occurrence and development of hepatocellular carcinoma. Signal transduction and transcriptional activator-6 (STAT6) is highly expressed in various human cancers. Consistent evidence suggests that STAT6 predicts poor prognosis in patients with hepatocellular carcinoma (HCC) [55]. Xiao et al. [56] found that AR/miR-520f-3p/SOX9 signaling altered HCC cells by increasing cancer stem cell (CSC) population. Luo et al. believe that the mechanism of sorafenib combined with silencing activation of transcription factor 2 (ATF2) may be related to the activation of the TNF-*α*/JNK3 signal transduction [57]. LEN (a linear tyrosine kinase inhibitor) may be an important therapeutic method. The efficacy of locally advanced patients who underwent selective internal radiotherapy (SIRT) was similar to that of patients treated with sorafenib [58]. For patients with HCC who are not suitable for surgery and other local area treatments, we may consider central liver stereotactic whole body radiation therapy (SBRT). This treatment regimen produces a good therapeutic response to centrally located hepatocellular carcinoma (CL-HCC) with acceptable HBT (hepatobiliary toxicity) and LC (local control) [59].

There are some limitations in our manuscript. Firstly, although the key module and key genes were identified, no in vitro experiments were performed to validate the conclusions. Secondly, given the characteristics of the dataset we used, we could not explore the key genes by different stages. All the above would be performed in the future.

## 5. Conclusions

According to data mining and modularized research methods, we conducted a series of comprehensive and systematic analyses to reveal the underlying pathogenesis of hepatocellular carcinoma. According to the genetic characteristics of liver tissue, we explored the functional disorder module of hepatocellular carcinoma and revealed its pathogenesis and guided personalized care and treatment. It not only provides a new way for biologists and medical scientists to study hepatocellular carcinoma but also provides a valuable reference for its subsequent diagnostic treatment.

## Data Availability

The datasets used and/or analyzed during the current study are available from the corresponding author on reasonable request.

## Supplementary Materials

Supplementary 1 Table S1.4479 Hepatocellular carcinoma differential genes.

Supplementary 2 Table S2.36231 Functional enrichment results.

Supplementary 3 Table S3.356 ncRNA-Module target pairs.

Supplementary 4 Table S4.52 TF-Module control pairs.
