# Supplementary material for: Gene Expression Characteristics of Liver Tissue Reveal the Underlying Pathogenesis of Hepatocellular Carcinoma
Source: Biomed Res Int. 2021 Oct 4;2021:9458328. doi: 10.1155/2021/9458328 (PMC8506137; doi:10.1155/2021/9458328)
Supplement: Supplementary 1 — Table S1.4479 Hepatocellular carcinoma differential genes. [file 9458328.f1.docx]

| Table S1.4479 hepatocellular carcinoma differential genes |
| --- |
| DEG |
| CCK |
| RFPL2 |
| PRAMEF10 |
| MYH2 |
| FGF21 |
| CYP26A1 |
| RFPL1 |
| DACT2 |
| CGA |
| IP6K3 |
| IL20RA |
| LINC01235 |
| NAALADL2 |
| SCUBE1 |
| CDH15 |
| LINC00582 |
| CYP7A1 |
| GJB5 |
| CFAP221 |
| CHAC1 |
| GABRB3 |
| PPP1R3G |
| ISM1 |
| PACRG |
| FAM3B |
| ADAMTS18 |
| NALCN |
| FAM92B |
| RFPL3 |
| LINC00881 |
| PLPP4 |
| CYP26B1 |
| DDX43 |
| C6orf99 |
| OR10X1 |
| LPAR3 |
| CBLN2 |
| EMX2OS |
| LOC100505918 |
| TNFRSF17 |
| TENM4 |
| CLEC4F |
| GRM8 |
| HOXA13 |
| HOXD8 |
| AMPD1 |
| ALKAL1 |
| SNORD67 |
| CXCL9 |
| RAB25 |
| MZB1 |
| LOC389831 |
| IGSF10 |
| C11orf53 |
| FAM131B |
| TEX26 |
| SNAP25-AS1 |
| ESRP1 |
| NR4A2 |
| STMN2 |
| KCNK2 |
| RIBC2 |
| SCARA5 |
| PTCHD4 |
| FUT2 |
| DTHD1 |
| FAM19A2 |
| BAALC-AS1 |
| UNC80 |
| CNNM1 |
| SLC25A21 |
| CLDN10 |
| TOX3 |
| LINC00851 |
| IRF4 |
| COLCA2 |
| BPY2B |
| POU2AF1 |
| OXGR1 |
| LINC00271 |
| CD207 |
| ZG16B |
| QRFPR |
| TMC4 |
| LRFN5 |
| ENPP5 |
| CXCL10 |
| TENM2 |
| MRAP2 |
| SOST |
| CPB1 |
| PNOC |
| XK |
| LRRC1 |
| GDF6 |
| CLDN11 |
| IRX5 |
| KIF15 |
| PEBP4 |
| CLSPN |
| CALN1 |
| ULBP1 |
| KCNJ16 |
| MEX3B |
| SNHG18 |
| ARSF |
| TMEM130 |
| LRAT |
| WNT10A |
| KAZALD1 |
| OSR2 |
| CNTNAP3B |
| CBLN4 |
| A4GNT |
| CEACAM7 |
| ZNF204P |
| PCDH19 |
| IL34 |
| WFDC2 |
| DLGAP5 |
| PRKN |
| UGT3A2 |
| LOC100240734 |
| IL5RA |
| TFF3 |
| AP1M2 |
| TAC3 |
| GPR83 |
| CALB1 |
| FRAS1 |
| AK8 |
| SFRP5 |
| AHRR |
| OOSP2 |
| CUBN |
| OR4A16 |
| RHBDL3 |
| EPN3 |
| DIRAS2 |
| PGK2 |
| SNAP25 |
| KLK1 |
| PRPH |
| CASQ1 |
| ANXA8L1 |
| LRRC69 |
| UPK1B |
| VSIG2 |
| CALB2 |
| MB |
| CLSTN2 |
| TMPRSS3 |
| PRAMEF4 |
| COL22A1 |
| PGLYRP3 |
| ITGBL1 |
| SPATA45 |
| TP53TG3D |
| RNF157-AS1 |
| OR2M4 |
| AWAT2 |
| STAC |
| PRAMEF15 |
| EFCAB6 |
| PLCXD3 |
| LRMDA |
| MUC21 |
| TMEM178B |
| ZPLD1 |
| LINC00971 |
| PRR15L |
| PNLIPRP2 |
| FGF19 |
| CCDC171 |
| FERMT1 |
| CLIP3 |
| CYS1 |
| SCUBE3 |
| CD27 |
| C10orf113 |
| VEGFD |
| PCDHA3 |
| ANO4 |
| ENTPD3 |
| NXPE4 |
| DUXA |
| ERCC6L |
| MTNR1A |
| PTGES |
| GPRC6A |
| OTX2 |
| PRMT6 |
| NEK10 |
| E2F2 |
| BHLHE22 |
| RASGRF1 |
| GABRA2 |
| FLVCR1-DT |
| IGDCC4 |
| CYSLTR2 |
| VLDLR-AS1 |
| EPCAM |
| LRRC7 |
| WNT10B |
| SCOC-AS1 |
| MKRN2OS |
| GPC6 |
| FAM167A |
| LINC01686 |
| LINC00319 |
| CHST4 |
| PDGFD |
| PCDH9 |
| RAPGEF3 |
| RASSF9 |
| LINC00207 |
| OPRM1 |
| DMBT1 |
| CABS1 |
| ANXA13 |
| ODAM |
| BFSP2 |
| SSPN |
| SLC34A2 |
| SLC26A4 |
| C17orf100 |
| PF4V1 |
| LINC00898 |
| FCER1A |
| NRCAM |
| BSPRY |
| INA |
| SH3PXD2A-AS1 |
| VAT1L |
| ASB2 |
| GABRR1 |
| PURPL |
| SPINK13 |
| SGCZ |
| SLCO2A1 |
| CPXM2 |
| NRXN3 |
| FSD1L |
| SPATS1 |
| MKI67 |
| PSMA8 |
| TAS2R4 |
| MMP12 |
| CTNNA3 |
| TNFRSF13B |
| GATA5 |
| LAMP3 |
| LEP |
| C5orf58 |
| AURKA |
| RANBP3L |
| CDKN2A |
| MEI1 |
| PQLC2L |
| RBMY2FP |
| SOX9 |
| ST8SIA6-AS1 |
| COCH |
| IQCA1 |
| ATP13A4 |
| IL2 |
| DKK3 |
| ANLN |
| PRAMEF5 |
| PRSS56 |
| ESRRG |
| GRAP2 |
| KIF18A |
| MS4A8 |
| JCHAIN |
| TUBBP5 |
| ADGRB3 |
| SEMA3D |
| ACSM1 |
| PIGR |
| ZNF827 |
| VEPH1 |
| SULT1C2 |
| SYT8 |
| NPTX1 |
| FCRL5 |
| TPX2 |
| FMOD |
| DNAH7 |
| SPRR2A |
| SMYD3 |
| FAM83B |
| DKK4 |
| LRCH2 |
| CCDC146 |
| LOC100192426 |
| AVPI1 |
| CNTNAP2 |
| NLGN1 |
| UHRF1 |
| ALKAL2 |
| C16orf74 |
| TSHZ2 |
| ARHGEF28 |
| CYP4F22 |
| RSPO1 |
| ARHGAP8 |
| CA12 |
| SCN2A |
| LINC02275 |
| LOC401463 |
| IMMP2L |
| IGF2BP1 |
| SLC26A5 |
| FAM153B |
| PDZK1IP1 |
| COL21A1 |
| SSTR2 |
| RFTN2 |
| OR6M1 |
| LOC283710 |
| CLLU1OS |
| HBG1 |
| IL4I1 |
| SCNN1B |
| VANGL2 |
| LRRC4C |
| LAX1 |
| HAVCR1 |
| IGFBP6 |
| MYOC |
| KCNN3 |
| ZNF683 |
| ANKRD29 |
| BBS9 |
| DLX6-AS1 |
| NKAIN3 |
| DACT3-AS1 |
| OOEP |
| SPOCD1 |
| PYGO1 |
| ANKRD37 |
| KCNK4 |
| PIP5K1B |
| GTSE1 |
| GSN |
| C9orf66 |
| ZNF93 |
| INHA |
| LAMP5 |
| CLEC2B |
| ZNF665 |
| HOXA3 |
| NPW |
| TLL1 |
| TRPM8 |
| ST14 |
| PCP4 |
| LINC01133 |
| NOP14-AS1 |
| TP53TG3HP |
| LTBP2 |
| HOXB3 |
| ANKUB1 |
| LINC01548 |
| CD79A |
| PPP1R14C |
| FDCSP |
| MYLK-AS1 |
| C21orf62 |
| C17orf102 |
| TEX11 |
| C6orf222 |
| SOWAHB |
| CYP4B1 |
| RFLNA |
| LINC00642 |
| CEP112 |
| DPT |
| NME5 |
| KANK4 |
| RYR3 |
| SATB2 |
| MCF2 |
| TYRP1 |
| ISLR |
| ERC2 |
| CKMT2 |
| CD1B |
| FLJ42102 |
| MKX |
| NCR3 |
| SEMA5A |
| ZC3H12B |
| PCLO |
| DLG3 |
| LRRTM4 |
| PTTG1 |
| LOC100506688 |
| LAYN |
| HHAT |
| GSDME |
| CDKL2 |
| HCAR1 |
| C8orf87 |
| IGLL5 |
| OR5B21 |
| ANKRD36BP2 |
| COPG2 |
| WDR11-AS1 |
| S100A14 |
| MMP2 |
| TMPRSS4 |
| GNG12-AS1 |
| PRKG1 |
| LPAR4 |
| GABRA5 |
| GLI3 |
| SELP |
| KIF23 |
| KCND1 |
| DNAJC6 |
| SPINT2 |
| ATP6V1B1 |
| RSPH1 |
| CCDC30 |
| DPH6-DT |
| FER1L4 |
| ZG16 |
| CTLA4 |
| UNC5B |
| CPE |
| P2RY10 |
| HMCN1 |
| SPAG4 |
| RGS1 |
| TEPP |
| WIPF3 |
| APCDD1L-DT |
| TMC7 |
| TYR |
| KATNBL1P6 |
| GPRIN2 |
| NPFFR2 |
| ENDOU |
| KLC3 |
| SH2D3A |
| KL |
| GDF2 |
| ADAMTS10 |
| LOC100507377 |
| PPP1R1B |
| CDRT1 |
| AIM2 |
| SSTR5 |
| FAM66D |
| RNF157 |
| ANKRD7 |
| NEXMIF |
| CACNG6 |
| COL20A1 |
| CD226 |
| OLFML3 |
| ACSL6 |
| IZUMO1R |
| GRIA3 |
| NLGN3 |
| FST |
| LARP6 |
| FHIT |
| PHACTR3 |
| FGF20 |
| PDZD4 |
| MYOM3 |
| STAP1 |
| PLAU |
| DPRXP4 |
| PTTG2 |
| IGFL2 |
| SLC44A3 |
| PLEKHB1 |
| SMPD3 |
| FZD3 |
| C7 |
| KCNA2 |
| RRAD |
| NEK11 |
| C7orf31 |
| NLRP10 |
| RALGPS1 |
| LINC00917 |
| RAD54L |
| LOC100288637 |
| FOXF1 |
| PRR11 |
| COL4A5 |
| LDLRAD2 |
| PGM5-AS1 |
| CLEC10A |
| SHISA2 |
| UCMA |
| BMP3 |
| SLC25A26 |
| CNTN3 |
| DOC2A |
| SLC28A3 |
| LTBP1 |
| CXCL14 |
| EPHA3 |
| CCDC74B |
| SEZ6L2 |
| CST6 |
| FAM110C |
| ABI3BP |
| ICOS |
| KLHL33 |
| CD8A |
| PCED1B |
| CD28 |
| ADGRF1 |
| CRIM1-DT |
| COL23A1 |
| DSC1 |
| HOXD1 |
| VGLL3 |
| TOB1 |
| ZIC5 |
| SCG3 |
| TRHR |
| DCDC2 |
| CCR10 |
| DGKI |
| CHODL |
| UTS2B |
| MUC15 |
| HTR1F |
| C3orf67 |
| MMP16 |
| KCNK6 |
| APOBEC3G |
| MST1L |
| CACNA1A |
| ADGRG5 |
| NXF2 |
| CIB4 |
| SOX10 |
| COLGALT2 |
| CHRDL1 |
| PDE4D |
| SLAMF7 |
| CNR1 |
| RLN2 |
| RASGRP1 |
| FAM182A |
| MEGF6 |
| OR4A47 |
| NCCRP1 |
| CEMIP |
| ST8SIA1 |
| UNC79 |
| LINC01060 |
| PDGFRA |
| NPHP1 |
| LOC374443 |
| ACVR1C |
| PIP4P2 |
| C4orf33 |
| MINAR1 |
| FAM19A1 |
| SAMD9L |
| CCDC3 |
| OR1L6 |
| CD24 |
| TRABD |
| SIX2 |
| NEK5 |
| CBX4 |
| SGCG |
| RHOH |
| LINC01512 |
| TMEM98 |
| GDF15 |
| EPHB3 |
| 11-Mar |
| LOC100506474 |
| OR1L3 |
| ZMAT3 |
| CACHD1 |
| IL22 |
| TTYH1 |
| FANCI |
| UAP1L1 |
| MAEL |
| SLC25A25 |
| PLEKHH2 |
| RD3 |
| PAMR1 |
| TEX38 |
| PACSIN1 |
| CCNE1 |
| SEPT1 |
| PLD4 |
| TMEM254-AS1 |
| PTPRD |
| RBP2 |
| DZIP1 |
| ASIC2 |
| SLC6A9 |
| APOBEC4 |
| SH3YL1 |
| C20orf78 |
| GLT8D2 |
| CD8B |
| PAQR5 |
| SAMD14 |
| OR1I1 |
| CARTPT |
| KCNA1 |
| GGT8P |
| ZNF90 |
| JHY |
| COX6A2 |
| FADS6 |
| GRB7 |
| CILP |
| APOOP5 |
| KCNA3 |
| VPREB3 |
| UBD |
| ZRANB3 |
| OR51B5 |
| SHISAL2A |
| ASPN |
| BIRC5 |
| LRP2BP |
| ITIH5 |
| SLC27A6 |
| NTM |
| SRY |
| RSAD2 |
| OAS3 |
| ZSCAN31 |
| RBP1 |
| C14orf177 |
| ADAMTSL3 |
| C1QL4 |
| CRTAP |
| PCYT1B |
| RARB |
| LAMA3 |
| SPICE1 |
| LINC02171 |
| NOMO1 |
| GLB1L2 |
| LMCD1-AS1 |
| EPHA7 |
| CLVS1 |
| ADAMTS12 |
| PBX1 |
| MFAP4 |
| C8orf34 |
| TIGIT |
| KIAA1328 |
| PRIMA1 |
| PSG3 |
| B4GALNT3 |
| CLIC6 |
| NXPH3 |
| RXRG |
| SLC1A2 |
| NEU2 |
| SFRP1 |
| RIMS2 |
| PATL2 |
| GRID1-AS1 |
| GRM7 |
| PLCH1 |
| SEL1L2 |
| OTOP1 |
| BATF2 |
| LINC00696 |
| SPHKAP |
| JSRP1 |
| PCDHB18P |
| KIT |
| POU4F1 |
| VLDLR |
| RIMS1 |
| NGB |
| ARL10 |
| DLGAP2 |
| OR8K5 |
| SPATS2 |
| MIR221 |
| GZMH |
| CHIA |
| FAXC |
| CLEC1A |
| TENM3-AS1 |
| C2orf81 |
| XXYLT1 |
| GATA1 |
| NFASC |
| CCDC149 |
| FZD1 |
| MATN2 |
| TRIM22 |
| UBA7 |
| MXRA8 |
| LRRIQ1 |
| TLE2 |
| PCDHB9 |
| ARHGAP22 |
| NPAS3 |
| TLE6 |
| NR2F1-AS1 |
| CRTAM |
| NUCB2 |
| P2RY8 |
| MAMDC2-AS1 |
| NEK8 |
| SNORD114-24 |
| STK32B |
| HLA-DOB |
| AQP4 |
| NBPF22P |
| PXDNL |
| HNF1B |
| SLC47A1 |
| FBXO36 |
| KERA |
| B3GALT5-AS1 |
| CXCL17 |
| GP1BA |
| PP12613 |
| IFT57 |
| TPTE2P1 |
| FMR1-AS1 |
| INKA2-AS1 |
| DEFA6 |
| RCVRN |
| PRICKLE1 |
| KLF11 |
| GALNT18 |
| NPS |
| CLLU1 |
| NSG2 |
| GADL1 |
| HIST1H2AC |
| KHDC1 |
| MROH8 |
| PIK3CG |
| KLHDC7B |
| MEIS3 |
| IHH |
| TMEM47 |
| GPR137 |
| NBAS |
| TMEM196 |
| ASTN2 |
| SLC26A7 |
| GLIS2 |
| TRIM58 |
| TARM1 |
| OR8D4 |
| PTPN7 |
| TLCD2 |
| SNORD62A |
| GOT1L1 |
| KCTD16 |
| SAMD15 |
| DEFB114 |
| VAX1 |
| MS4A6E |
| C6orf118 |
| FN1 |
| LINC02381 |
| FXYD2 |
| C17orf99 |
| ARHGEF38 |
| TESMIN |
| SDK2 |
| KRT72 |
| CIP2A |
| PAPLN |
| FKBP11 |
| ZNF804A |
| TNN |
| ARRB1 |
| HIST1H2BB |
| SLC49A3 |
| NCALD |
| CCDC105 |
| TPK1 |
| LRBA |
| TUSC3 |
| TTTY12 |
| PCDHB17P |
| DCN |
| MAGEC2 |
| AMIGO2 |
| ST6GALNAC4 |
| WDPCP |
| CHRNA6 |
| HIST1H2BM |
| AQP1 |
| SCD |
| FANCD2 |
| PTPRM |
| MPND |
| TPTE2 |
| DEFB135 |
| LAMB2 |
| RAD51B |
| PLOD1 |
| EPHB6 |
| BLOC1S2 |
| ZNF135 |
| C2orf91 |
| FBLN7 |
| MAN2B2 |
| BCAS3 |
| ARL14EPL |
| HIST1H2BI |
| SIL1 |
| PLEKHS1 |
| ADAMTS16 |
| C9orf43 |
| SMIM1 |
| ACAD10 |
| ZMIZ1-AS1 |
| NXF5 |
| KIRREL3 |
| HIST1H2BK |
| AGRN |
| CDHR5 |
| TGM1 |
| VWCE |
| CBR3 |
| LINC00607 |
| PURG |
| HOXA2 |
| XRRA1 |
| LINC00654 |
| HIST1H3B |
| POLR1E |
| HACD1 |
| MXRA5 |
| XAF1 |
| BEND6 |
| ZNF555 |
| PTGIR |
| ERAP1 |
| HIST1H2BL |
| FBXL19-AS1 |
| SLFN11 |
| SDC2 |
| ANO10 |
| FOCAD |
| ACAP1 |
| MAB21L2 |
| HIST1H2BD |
| MAP4K1 |
| GRIN2A |
| E2F7 |
| TRPV2 |
| SIDT1 |
| MRAS |
| CATSPERZ |
| LOC100132077 |
| BARD1 |
| NGFR |
| ORAI1 |
| ARID3A |
| C19orf18 |
| IGSF5 |
| LINC00840 |
| GYPC |
| LINC01502 |
| FAM162B |
| DPP4 |
| SHISA4 |
| MIR9-3HG |
| HIST1H2BO |
| SEPT6 |
| HIST1H2BH |
| MIPOL1 |
| SMAD7 |
| CEP89 |
| ARVCF |
| SLC39A8 |
| IFI44L |
| MCUB |
| CD48 |
| ANXA4 |
| MUCL3 |
| MYL6B |
| ZNF670 |
| MYOM1 |
| FMO5 |
| HKR1 |
| HIST1H2BG |
| EFCC1 |
| ARHGEF3 |
| FAM71E1 |
| GM2A |
| ID3 |
| ST5 |
| ZNF782 |
| BSG |
| GOLGA8F |
| SLC3A2 |
| TOMM34 |
| LOC554206 |
| RASGRP2 |
| U2AF2 |
| MTMR10 |
| ATP6V0A1 |
| GTPBP10 |
| GOLGA6L1 |
| LIMD1 |
| MYO19 |
| FIGNL2 |
| LINC01118 |
| EBI3 |
| TLR6 |
| UTP6 |
| ZRANB2 |
| ENTPD4 |
| GPC1 |
| ZNF326 |
| NCBP2 |
| PDCD11 |
| CFAP126 |
| SNORD95 |
| LOC338963 |
| ZNF587B |
| DDX52 |
| TMEM105 |
| PNISR |
| FNDC3B |
| C8orf74 |
| RNASEH2C |
| ETNK2 |
| RTP5 |
| C10orf111 |
| TTC14 |
| CRIP2 |
| PSMG4 |
| ACACA |
| SEH1L |
| INSL3 |
| VSIG10L |
| UBR2 |
| DDX51 |
| TIFA |
| LY6G5C |
| SPACA5 |
| CCZ1P-OR7E38P |
| PPARGC1B |
| CFAP73 |
| FMO4 |
| MPIG6B |
| ZNF485 |
| A2MP1 |
| SSC4D |
| POFUT1 |
| TXK |
| NIPSNAP3B |
| ALKBH2 |
| GFOD2 |
| PSMA1 |
| PM20D1 |
| ZNRF2P1 |
| ZNRF4 |
| SMG5 |
| SLFN14 |
| IRF6 |
| SNORA71E |
| SSH1 |
| RADX |
| BEX5 |
| C1orf226 |
| UNC5A |
| ZNF217 |
| OR8B8 |
| AQP7P1 |
| LINC00652 |
| DHX33 |
| LINC00648 |
| SNORA31 |
| LINC00485 |
| ZBTB7A |
| CAMK2N2 |
| TXNRD3NB |
| KHDC4 |
| LINC01239 |
| METTL7B |
| CYP46A1 |
| PPP1R27 |
| PLEKHA4 |
| TSEN15 |
| LOC100507389 |
| ZNF396 |
| FGD5 |
| PFN1P2 |
| NUDT14 |
| ADGRF5 |
| DHCR7 |
| MANCR |
| PAGE2B |
| FAN1 |
| ARL17A |
| HIST1H4H |
| KCNAB1 |
| ZNF708 |
| IL6R |
| BTBD11 |
| GDI1 |
| KRTAP10-3 |
| BTNL9 |
| ZC3H8 |
| HIST2H4B |
| HIST1H4F |
| ITGB2-AS1 |
| FOXI1 |
| KIR3DL1 |
| SNHG10 |
| PAGE2 |
| MELTF |
| RASGEF1B |
| CLEC12B |
| ACVR2B |
| SNHG20 |
| DAPK2 |
| PPM1E |
| LINC00977 |
| PRSS54 |
| GTF2H2C_2 |
| MSANTD3 |
| LRRK2 |
| SPAG5-AS1 |
| SIRPD |
| RGPD6 |
| ALOX12P2 |
| ZCCHC13 |
| GLDC |
| TDRD6 |
| LOC646938 |
| DTX1 |
| CDYL2 |
| ZNF540 |
| WDR3 |
| KLHL41 |
| PRICKLE4 |
| DCSTAMP |
| LINGO4 |
| LOC644656 |
| SNORD25 |
| CSNK1A1P1 |
| POU4F3 |
| CCDC166 |
| COBL |
| TTC22 |
| SNORA8 |
| SMARCD2 |
| AMY1C |
| C3AR1 |
| CFAP97D1 |
| LCN8 |
| LINC01268 |
| FAM155B |
| RNF103 |
| PKD2L1 |
| ALPK3 |
| S100A5 |
| GPR162 |
| KBTBD12 |
| OR7E14P |
| FLJ42351 |
| SNORA60 |
| SNORD114-26 |
| OR2AE1 |
| RBP7 |
| STEAP2 |
| RPL27A |
| BCL2L11 |
| NR6A1 |
| PAX6-AS1 |
| OR7E24 |
| OR7E12P |
| ADAM20 |
| KLK12 |
| TTN-AS1 |
| ZRANB2-AS1 |
| LINC01015 |
| SNORD16 |
| RNU12 |
| OR7E156P |
| SNHG3 |
| LINC00638 |
| APBB3 |
| SMG1P7 |
| ASB9 |
| GSDMA |
| ITGA2B |
| FLJ37201 |
| GUSBP5 |
| NUDT8 |
| LCE2D |
| PCDHGB4 |
| SNHG9 |
| LOC100505912 |
| SIGLEC5 |
| RND2 |
| SNORA18 |
| SNORA20 |
| KRT35 |
| LINC00115 |
| PNLDC1 |
| SLC26A8 |
| ASB9P1 |
| MEIG1 |
| PVR |
| SCGB3A2 |
| DTX4 |
| BHLHE40-AS1 |
| BSN-DT |
| ZNF202 |
| TUBB1 |
| CCL3 |
| PFKFB1 |
| HIST1H2BA |
| EPM2AIP1 |
| ASIP |
| TMED6 |
| XPNPEP2 |
| MEG8 |
| LOC100128993 |
| APBA1 |
| DDX25 |
| UPP1 |
| TTLL12 |
| LOC652276 |
| POU6F2 |
| ITGB3 |
| CRYBB2P1 |
| TFRC |
| C2CD4B |
| KCNN1 |
| MIR4453HG |
| DISP2 |
| ZP2 |
| TENM1 |
| BEND3P3 |
| KIR2DS3 |
| PCSK1 |
| SNORA61 |
| ABCC11 |
| OGDH |
| CYP4Z1 |
| DEFA5 |
| SNORA63 |
| LINC00640 |
| KRT15 |
| FOXP3 |
| NUDT10 |
| CCL3L3 |
| LINC00189 |
| MCTS2P |
| SNORD72 |
| C3orf84 |
| CCL18 |
| LOC646903 |
| SNORA24 |
| WFIKKN1 |
| CALCA |
| SNORD115-2 |
| SNORD45B |
| FAM169B |
| LDLRAD4-AS1 |
| ESM1 |
| NANOG |
| LINC01252 |
| MROH7 |
| SOX12 |
| RNASE11 |
| CCL11 |
| CEP83-DT |
| LINC00862 |
| SLC22A2 |
| LINC00222 |
| PI15 |
| SLC1A3 |
| SLC25A27 |
| NLRP2 |
| SALL4 |
| ZFP57 |
| INS-IGF2 |
| RPPH1 |
| VSTM5 |
| CRYBB2 |
| FAM184B |
| DPPA4 |
| FBN2 |
| TNFSF18 |
| SFRP2 |
| CPNE6 |
| TAS2R40 |
| TCAF2 |
| NOS1AP |
| C17orf98 |
| ARC |
| LOC154761 |
| APOA4 |
| ADAMTS14 |
| SNORA51 |
| SSUH2 |
| CYP27C1 |
| GPR88 |
| UMODL1-AS1 |
| EPHA1-AS1 |
| SLITRK3 |
| SLC51B |
| SMIM32 |
| THBS4 |
| AQP10 |
| RAB3B |
| SFRP4 |
| EPS8L3 |
| CST2 |
| APLN |
| GSTT1 |
| GPR19 |
| DNER |
| EEF1A2 |
| NXPH4 |
| REEP2 |
| TMEM151A |
| CDT1 |
| SPOCK1 |
| FAM19A5 |
| E2F8 |
| CHRNB2 |
| GNG4 |
| COL10A1 |
| SCGN |
| GABBR2 |
| MUC13 |
| MSMB |
| TTK |
| KCNC1 |
| MCM10 |
| HJURP |
| CENPM |
| KIF20A |
| FAP |
| NANOS1 |
| EPHX4 |
| IQGAP3 |
| RTKN2 |
| WNT4 |
| TOP2A |
| HIGD1B |
| PTK7 |
| SBK1 |
| CDC25C |
| CTHRC1 |
| UBE2C |
| RGS9 |
| RAB6B |
| NEK2 |
| MYT1 |
| CEP55 |
| FBXO43 |
| EDIL3 |
| PBK |
| BEX2 |
| RIPPLY2 |
| MBOAT4 |
| COL15A1 |
| CENPA |
| TROAP |
| KIFC1 |
| CST1 |
| GAREM2 |
| PLCE1 |
| MELK |
| GPC3 |
| TRIM9 |
| MAPK8IP2 |
| CENPF |
| PAQR4 |
| CD200 |
| RSPH14 |
| CDCA5 |
| FOXS1 |
| MAPT |
| SEZ6 |
| TMEM198 |
| RAB3C |
| SYP |
| BTBD17 |
| BUB1 |
| CELSR3 |
| NEB |
| EPPK1 |
| UBE2T |
| PLXDC1 |
| TUBB3 |
| PTTG3P |
| CDCA2 |
| BFSP1 |
| SEPT3 |
| SIX4 |
| MELTF-AS1 |
| ADCYAP1 |
| SAPCD2 |
| OVOL2 |
| SKA3 |
| SALL2 |
| ERN2 |
| GNAZ |
| TOB2P1 |
| MMP1 |
| ARHGEF39 |
| COL8A1 |
| BEX1 |
| CDC45 |
| MYBL2 |
| CKMT1A |
| TCAM1P |
| LINC00673 |
| CTNND2 |
| SPC25 |
| PNMA8A |
| TRPM5 |
| SPINT1 |
| CTSV |
| DUOX1 |
| CDC25A |
| SEC14L5 |
| GAL3ST1 |
| DAND5 |
| DIAPH3 |
| CCNB1 |
| KIF2C |
| MMP11 |
| FBXL21 |
| SHCBP1 |
| EXO1 |
| DTNA |
| KIF14 |
| SUSD4 |
| CDCA7 |
| CCNB2 |
| MAP1A |
| TMEM266 |
| MATN3 |
| TLDC2 |
| GINS1 |
| TRIM45 |
| KIF4A |
| S100A3 |
| DKK2 |
| NAT14 |
| LINC01116 |
| CDKN3 |
| CDKN2B-AS1 |
| B4GALNT1 |
| TMEM270 |
| UNC5B-AS1 |
| C3orf80 |
| CDCA3 |
| PRC1 |
| NPTX2 |
| ITGA2 |
| BCAS4 |
| ZNF534 |
| MIMT1 |
| SFTA1P |
| TRPC4 |
| ZNF454 |
| NOX4 |
| CFAP43 |
| COMP |
| SERTAD4 |
| PRR19 |
| FOXD2 |
| CCNA2 |
| CKAP2L |
| PDE4C |
| COL1A1 |
| RAB3A |
| PIFO |
| KIAA1549 |
| FOXE1 |
| H2AFY2 |
| DTL |
| NUF2 |
| TRIP13 |
| ITPR3 |
| FAM81A |
| ASF1B |
| MRPL23-AS1 |
| BCL9 |
| DIPK2B |
| FOXL1 |
| PCDHB10 |
| CENPK |
| ORC6 |
| CCNF |
| AFF3 |
| TMSB15A |
| SEPT5 |
| C2orf27A |
| H2BFM |
| ABCA3 |
| EBF3 |
| HELLS |
| TCF19 |
| ANKRD65 |
| DEPDC1 |
| CHAF1B |
| SULF1 |
| B3GNT3 |
| WDR76 |
| LINC02159 |
| KIF24 |
| BUB1B |
| RAB9B |
| NRSN2 |
| TMSB15B |
| JAG2 |
| SPATA17 |
| CADPS |
| HIST1H2AI |
| CHEK1 |
| LOXL1 |
| SPC24 |
| CENPE |
| GPSM2 |
| NDC80 |
| SYNGR1 |
| NT5DC2 |
| TNNI3K |
| CDH13 |
| NPTXR |
| CSPG5 |
| GDAP1 |
| SAPCD1 |
| BARX2 |
| PRRX1 |
| SGO2 |
| ETV4 |
| RIMKLA |
| ACOXL |
| BICDL1 |
| SERTAD4-AS1 |
| RNFT2 |
| GLI1 |
| PRND |
| DYDC1 |
| EGLN3 |
| ORC1 |
| CKB |
| EFHC1 |
| HIST1H2AL |
| ADAM22 |
| PLCD3 |
| LUZP2 |
| NPM2 |
| FAM24B |
| CBX2 |
| CDH11 |
| C2orf15 |
| SLCO1C1 |
| GUCA2A |
| EBF1 |
| SMC1B |
| MN1 |
| MND1 |
| THY1 |
| LEF1-AS1 |
| CABYR |
| C3orf70 |
| PRR16 |
| BRSK2 |
| GJC1 |
| JAM3 |
| SPATC1L |
| LINC00312 |
| CUZD1 |
| NPNT |
| TMEM145 |
| TRIM59 |
| LEF1 |
| PMCHL1 |
| C11orf80 |
| LINC00665 |
| CHST3 |
| NUSAP1 |
| CACNA1D |
| JPH1 |
| RNF144A |
| CDK1 |
| H2BFXP |
| LOC100507554 |
| CDH6 |
| NDUFA4L2 |
| MTFR2 |
| RIMBP3 |
| OLFML2B |
| FHDC1 |
| HTRA3 |
| TIGD4 |
| MMP24 |
| LRFN1 |
| PLVAP |
| SRSF12 |
| DYDC2 |
| PDGFRL |
| WNK2 |
| IQCC |
| MFSD6 |
| PDE9A |
| CAVIN4 |
| MFGE8 |
| PARPBP |
| CNTNAP1 |
| TNFRSF4 |
| GALNT12 |
| ITGA3 |
| FATE1 |
| DLK2 |
| ESCO2 |
| TGFB2 |
| MEX3A |
| SLC7A5 |
| IL17D |
| ZFP82 |
| MSI1 |
| LOC400710 |
| ROR1 |
| MCM3AP-AS1 |
| FBXO39 |
| HDAC5 |
| PCSK1N |
| SGSM1 |
| JCAD |
| HASPIN |
| CNFN |
| TEDC2 |
| OBSL1 |
| RDM1 |
| RCAN3 |
| EBF2 |
| KIF11 |
| GRID1 |
| SNHG28 |
| ASPM |
| CLUL1 |
| OLFML2A |
| ZBED8 |
| NANOS3 |
| STIL |
| NPAS2 |
| RAB11FIP4 |
| LOXL2 |
| CIB2 |
| TMEM246 |
| STMN3 |
| MMP14 |
| CREB3L1 |
| SPINDOC |
| ANKRD34A |
| OR51E1 |
| KRBA1 |
| IPO9 |
| FGF1 |
| ZFP14 |
| ENPP2 |
| LURAP1 |
| STXBP6 |
| CACNA1B |
| PABPC4L |
| CENPL |
| PRSS30P |
| ATP6V0D2 |
| SEMA5B |
| ATP10A |
| SYCE2 |
| SPATA33 |
| INPP5J |
| TTLL1 |
| C18orf54 |
| LPL |
| THBS2 |
| LOC100506411 |
| AKR1E2 |
| PMCH |
| MMS22L |
| WDR62 |
| CD34 |
| PTPN14 |
| ASIC1 |
| SH3PXD2B |
| AURKB |
| CDIPTOSP |
| LOC284412 |
| RASD2 |
| LINC00511 |
| SLC29A4 |
| FOXF2 |
| FFAR4 |
| HID1 |
| CEP41 |
| GPX8 |
| NCAPG |
| COL5A1 |
| NOV |
| ANKS6 |
| PIMREG |
| C20orf96 |
| SLIT2 |
| HOXD9 |
| MDK |
| TMEM200A |
| RAB34 |
| LMCD1 |
| PEG10 |
| HEYL |
| NRM |
| PTP4A3 |
| LAMC1 |
| RHEX |
| GP1BB |
| TET1 |
| NETO2 |
| MIR600HG |
| AFAP1 |
| RAD51AP1 |
| RACGAP1 |
| KREMEN2 |
| SLC1A4 |
| LAMB2P1 |
| MSANTD3-TMEFF1 |
| OSBPL7 |
| CORIN |
| IBSP |
| SCAMP5 |
| B3GALNT1 |
| ZSWIM5 |
| ARMC9 |
| OXTR |
| CDCA8 |
| AZIN2 |
| GLS |
| TMEM237 |
| MFSD4A |
| NOTCH3 |
| AURKAP1 |
| C1QTNF6 |
| CENPH |
| PCLAF |
| ARL3 |
| KMT5C |
| MAD2L1 |
| TNFSF4 |
| DLL3 |
| CCDC102B |
| MAP1B |
| HIST1H2BF |
| MSMP |
| C12orf75 |
| KRT86 |
| P3H4 |
| CACNB3 |
| ELOVL7 |
| TMCO3 |
| MYO5C |
| GJA10 |
| LYPD1 |
| ENOX1 |
| EVA1B |
| RABL6 |
| NFATC4 |
| SGO1 |
| ARHGEF16 |
| SRC |
| JMJD1C-AS1 |
| LINC00515 |
| FANCB |
| SPDL1 |
| P4HA2 |
| TCTN2 |
| ZWINT |
| ARHGAP11B |
| BOC |
| DDX11-AS1 |
| LRRC49 |
| PCYOX1L |
| CENPW |
| FBXO16 |
| PRUNE1 |
| UBE2S |
| ESYT3 |
| STXBP4 |
| TRNP1 |
| HTR2A |
| TYRO3 |
| OSBP2 |
| RRM2 |
| SPEF2 |
| MAB21L4 |
| ECT2 |
| ATOH7 |
| OTUD7A |
| NT5M |
| PLK4 |
| FRZB |
| RNF24 |
| DPCD |
| COL3A1 |
| NCS1 |
| CLGN |
| DEFB131A |
| ULK4P1 |
| ZFP30 |
| KCNIP3 |
| BPTF |
| LRRC23 |
| THAP8 |
| PLP2 |
| OPHN1 |
| S100A2 |
| UACA |
| ABLIM2 |
| STX1A |
| CASTOR3 |
| MTBP |
| DBNDD2 |
| PHF19 |
| CHST1 |
| PKDCC |
| C2CD4C |
| ADAMTS6 |
| MFSD4A-AS1 |
| SLN |
| PGBD5 |
| CCDC28B |
| SULT4A1 |
| HES4 |
| FANCE |
| PRTFDC1 |
| RHOBTB2 |
| ZGRF1 |
| PAFAH1B3 |
| L3MBTL1 |
| LINC01140 |
| GIHCG |
| ZCCHC12 |
| EFNA3 |
| C5orf34 |
| TTYH2 |
| RECQL4 |
| LRP11 |
| TUFT1 |
| TMEM155 |
| LRRC75A |
| STX18-AS1 |
| ZFP2 |
| SMDT1 |
| TMEM8B |
| CADPS2 |
| CROCCP3 |
| TK1 |
| LY6H |
| LINC01963 |
| COL12A1 |
| COL17A1 |
| ZDHHC13 |
| SLC45A1 |
| CEP131 |
| OSBPL3 |
| CHD3 |
| COL1A2 |
| DBIL5P |
| TBC1D20 |
| SOX4 |
| ATP8A2 |
| CNPY4 |
| LOC100506472 |
| BLM |
| CHST10 |
| UBOX5 |
| KIF3A |
| C15orf39 |
| LZTS2 |
| C21orf58 |
| DSCC1 |
| HMGA1 |
| GATAD2B |
| LOC100499489 |
| WDHD1 |
| TUB |
| FOXM1 |
| TMEM108 |
| ESS2 |
| NHLH1 |
| PWWP2B |
| CYB561 |
| ZC2HC1A |
| BTRC |
| HEPH |
| FOXD4 |
| FLVCR1 |
| MYB |
| RARG |
| ATP6V1FNB |
| SLCO6A1 |
| AP3M2 |
| GPR137C |
| WSCD1 |
| CBFA2T2 |
| DLG5-AS1 |
| ZNF512 |
| SRI |
| NREP |
| SPIRE2 |
| TGM3 |
| ACTRT1 |
| AACS |
| NAA40 |
| FBXL18 |
| CCDC191 |
| FBLIM1 |
| LPCAT4 |
| FAM222A |
| PLCG1 |
| PTPDC1 |
| GINS4 |
| GPRIN1 |
| ICA1L |
| CD248 |
| RAD51 |
| LHX4-AS1 |
| FOXK1 |
| HENMT1 |
| FGD1 |
| CD109 |
| CCDC180 |
| TRAIP |
| COL4A1 |
| DNA2 |
| BRCA1 |
| FAM241B |
| CCDC102A |
| KIAA1614 |
| PLCB1 |
| JAG1 |
| ZNF660 |
| BTBD3 |
| METTL9 |
| ZNF503-AS2 |
| SYNE4 |
| PRRT2 |
| GRAMD1A |
| DNM3 |
| SCN4B |
| CDCA4 |
| KSR2 |
| HDAC7 |
| COL5A2 |
| LOC100134317 |
| AIFM2 |
| GBA |
| CASC15 |
| LOC100506123 |
| MIB1 |
| HEY1 |
| EHMT2 |
| RNASEH2A |
| TLN2 |
| PDGFB |
| CASQ2 |
| SLC26A2 |
| B3GNT5 |
| GGPS1 |
| FLYWCH1 |
| IFT52 |
| GTF2IRD1 |
| PLA2G1B |
| ARHGEF2 |
| CCDC24 |
| GOLGA2P10 |
| RHPN1 |
| PHYHIPL |
| ZNF532 |
| USP49 |
| ATAD5 |
| SH2B1 |
| ZNF251 |
| KNTC1 |
| BMPR1A |
| NOL4L |
| FBXL19 |
| PTOV1-AS1 |
| MIR4435-2HG |
| C19orf47 |
| LINC00471 |
| THSD7A |
| INE2 |
| PDGFA |
| SMG9 |
| BCAT2 |
| PGAP1 |
| TBC1D32 |
| SLC41A1 |
| PDGFRB |
| SELENOM |
| SESTD1 |
| DAGLA |
| ZNF678 |
| FMNL3 |
| CENPP |
| DCLK1 |
| SF3B4 |
| PIAS3 |
| CISD3 |
| CCDC103 |
| ZNF142 |
| ZSWIM4 |
| PYGB |
| TLCD1 |
| FANCG |
| SYCE1L |
| SLC44A5 |
| NUP210 |
| ACTG2 |
| FAM189B |
| BAHCC1 |
| C17orf53 |
| DBF4B |
| HIST1H2AB |
| SFXN3 |
| C4orf46 |
| ANKRD31 |
| PRR34-AS1 |
| HLA-V |
| ZNF300 |
| PKN1 |
| ARL2 |
| FBXL22 |
| DDR1 |
| CA5B |
| CEP250 |
| MDGA1 |
| MIEF2 |
| TMEM45B |
| MSH2 |
| ATP6V0E2-AS1 |
| ST7-AS1 |
| HIC2 |
| ZNF687 |
| TRAF2 |
| CENPO |
| NCAPD2 |
| PKMYT1 |
| TRAF5 |
| HSF2 |
| MINDY4 |
| NUDT17 |
| TBC1D22B |
| ABI2 |
| FLJ37035 |
| DSTYK |
| CDC7 |
| FBLN1 |
| GUSBP2 |
| ZNRF1 |
| ENAH |
| REM1 |
| ATP13A2 |
| ZNF74 |
| ZNF346 |
| CD2BP2 |
| NDRG3 |
| GPR176 |
| DUSP5P1 |
| DHRS13 |
| HSD11B1L |
| CERS3 |
| HACE1 |
| CLEC18B |
| TMEM107 |
| IFT80 |
| UNC119B |
| RNASEH1-AS1 |
| FBLN2 |
| CAMLG |
| PNMA1 |
| STRN |
| CHAF1A |
| KISS1 |
| LAPTM4B |
| SCAMP4 |
| FBXO27 |
| ATXN7L3 |
| AK9 |
| DBF4 |
| ZSWIM8-AS1 |
| GK5 |
| FKBP6 |
| ANKRD36B |
| FKBP10 |
| AEBP1 |
| DUSP9 |
| TTLL10 |
| SAC3D1 |
| TRIM46 |
| MED24 |
| PODXL |
| KLHL3 |
| FAT1 |
| PBXIP1 |
| MGP |
| CYB5RL |
| CLIC5 |
| FAM228B |
| IL12A |
| NCDN |
| NUDT1 |
| TCTEX1D2 |
| SVIL |
| DCLRE1B |
| FAM41C |
| UBN2 |
| PIGU |
| CABLES2 |
| LINC01012 |
| ERP44 |
| CEP162 |
| DNAJB5 |
| FAM169A |
| ZNF618 |
| STPG1 |
| KIAA1841 |
| COL16A1 |
| ZNF836 |
| RAB23 |
| WNT6 |
| SEPT8 |
| ARHGAP39 |
| ZNF286B |
| PPP2R3B |
| ZNF580 |
| HDAC11 |
| MORN4 |
| BCORL1 |
| CDPF1 |
| CSNK1E |
| PRR12 |
| C22orf23 |
| MYRF |
| CHTF18 |
| SHC3 |
| TARBP1 |
| C1orf74 |
| SLCO3A1 |
| PDC |
| GABRD |
| ELOB |
| COL4A2 |
| GYS1 |
| CDKL3 |
| FNDC1 |
| USP21 |
| RUFY2 |
| GLCE |
| HIST2H2AC |
| EHD2 |
| CCNE2 |
| TMEM101 |
| PRAF2 |
| ZNF581 |
| AP1S3 |
| ZNF608 |
| MTMR2 |
| AGAP1 |
| BRCA2 |
| SPATC1 |
| FLYWCH2 |
| C14orf93 |
| FLJ42627 |
| TSEN54 |
| MIER2 |
| H2AFX |
| POLA1 |
| GRM6 |
| CHML |
| CCDC97 |
| LLGL1 |
| TRAF1 |
| COL9A1 |
| IFT172 |
| SMAD2 |
| LAMA5 |
| PPP1R13L |
| CARMIL1 |
| TONSL |
| FAM219B |
| TAB1 |
| LRRC37A3 |
| PKN3 |
| GAGE2B |
| CHD6 |
| PACS2 |
| NCK2 |
| CDK19 |
| KCTD7 |
| CPXM1 |
| DVL3 |
| CHCHD6 |
| BMP8A |
| LAMA4 |
| CTIF |
| CDKL5 |
| LINC01704 |
| SLX4IP |
| PXYLP1 |
| LZTS3 |
| ZKSCAN3 |
| ZNF629 |
| INCENP |
| SNORD32B |
| POLQ |
| MCM6 |
| SFR1 |
| PLEKHH3 |
| ZBTB22 |
| ENTPD1 |
| PLEKHG2 |
| KAT7 |
| FGD6 |
| ZSCAN16 |
| KIF7 |
| PPCDC |
| ARHGEF11 |
| METTL27 |
| SNORD32A |
| PORCN |
| SLX4 |
| RFX8 |
| SMPD2 |
| NMB |
| PI4KB |
| ACTR1A |
| TBX2 |
| ARRDC2 |
| TULP3 |
| AGPAT1 |
| TIGD6 |
| ARMC2 |
| DDX46 |
| 9-Mar |
| HIST1H2AM |
| LAMB1 |
| LSM14B |
| LOC155060 |
| EXTL1 |
| FLJ43315 |
| WTIP |
| BAX |
| OLMALINC |
| VPS72 |
| CEACAM20 |
| MCF2L |
| HCG18 |
| NR2C2AP |
| DOCK6 |
| LDB1 |
| PALM |
| ZNF764 |
| LMNB2 |
| GTPBP2 |
| GJB4 |
| PPT2 |
| SLC25A41 |
| LOC100268168 |
| R3HDM1 |
| SLC35B4 |
| TP53TG5 |
| FHL3 |
| BPIFB2 |
| XRCC1 |
| BTF3L4 |
| EIF2A |
| HECTD2 |
| SMIM8 |
| ZNF850 |
| GNL1 |
| ZP3 |
| REEP4 |
| ZNF579 |
| DLG5 |
| FIZ1 |
| H2AFB2 |
| GSEC |
| NDE1 |
| ZNF792 |
| GIT1 |
| TMEM94 |
| CHST14 |
| INPP5F |
| JRK |
| HCFC1 |
| SMURF2P1-LRRC37BP1 |
| TPR |
| DENND6B |
| CPNE5 |
| BREA2 |
| NCOA5 |
| ZSWIM1 |
| TPM2 |
| NPM3 |
| OLA1 |
| SPNS1 |
| SP4 |
| CAPN11 |
| HCFC1R1 |
| ARID3B |
| ASB16 |
| FBXO17 |
| PSMC3IP |
| WASF1 |
| POC5 |
| PAK4 |
| PRXL2B |
| UXS1 |
| CHKB-DT |
| ADCK2 |
| PIGS |
| POPDC2 |
| B3GNTL1 |
| LRRC14 |
| PWWP2A |
| FOXD2-AS1 |
| CBX6 |
| BCR |
| KLHL22 |
| KCNH4 |
| LZTR1 |
| SASS6 |
| CMTM1 |
| ARFGAP1 |
| LCMT1 |
| TMEM52B |
| DDIT3 |
| BTN2A1 |
| EMID1 |
| ZNF292 |
| RHEBL1 |
| TTC13 |
| PRRC2A |
| ADD3-AS1 |
| FHAD1 |
| FBXO46 |
| UCN2 |
| CYTH3 |
| DAB2IP |
| C3orf62 |
| C1orf21 |
| NEU1 |
| SF3A2 |
| KIAA0895 |
| PRR36 |
| RASA4 |
| CD320 |
| TOR3A |
| FAM172A |
| DAXX |
| APC2 |
| APOBEC2 |
| RGL3 |
| MAPK3 |
| KCNF1 |
| CFAP157 |
| ZNF821 |
| POMT2 |
| SUCO |
| PROSER3 |
| PRCC |
| ITPRIPL2 |
| GPATCH1 |
| SPAG8 |
| CKAP5 |
| B3GAT2 |
| MAN1B1 |
| ZNF789 |
| MYH15 |
| HRAS |
| IFT81 |
| VPS45 |
| ABCB8 |
| CERS5 |
| EVI5L |
| H1FX-AS1 |
| BMT2 |
| MAML1 |
| LIG1 |
| POLL |
| ZNF775 |
| NDRG1 |
| PGS1 |
| ECPAS |
| SNX27 |
| LINC00960 |
| ATG4D |
| CAPRIN1 |
| AMN1 |
| DUSP28 |
| LRRC37B |
| ABCC10 |
| XPO5 |
| SPATA6 |
| ZNF473 |
| ZNF133 |
| CCNJL |
| C17orf82 |
| LRFN3 |
| DCK |
| SAMD4B |
| PRKD2 |
| TMEM79 |
| YEATS2 |
| CYB5B |
| CEP85 |
| ANKRD36 |
| NVL |
| DZIP3 |
| DCAF7 |
| LYG2 |
| HAUS6 |
| SSBP4 |
| HDAC2 |
| C9orf16 |
| MCM3 |
| NCOA6 |
| RBCK1 |
| ZNF48 |
| ZNF436 |
| SPIDR |
| ZCCHC3 |
| HIF1AN |
| ZSCAN9 |
| ZEB1-AS1 |
| ATG9A |
| BRF2 |
| SLC35B2 |
| VPS39 |
| PRR3 |
| STK36 |
| TCF3 |
| ZNF324B |
| DCAF16 |
| PCGF2 |
| RNF41 |
| NENF |
| RHBDF1 |
| DTNB |
| PIP4K2C |
| RAD9A |
| SNORD123 |
| BAZ2A |
| RAD54B |
| SRRM5 |
| RTL8A |
| CIZ1 |
| MEIOC |
| ARMC7 |
| CCSAP |
| ZNF606 |
| SLC41A3 |
| WASF2 |
| CX3CL1 |
| HSPA2 |
| LRRC37A6P |
| NUMA1 |
| CNOT6 |
| ARL15 |
| CCDC7 |
| ARHGAP33 |
| CADM4 |
| PRRG3 |
| LOC100129931 |
| BCOR |
| CABIN1 |
| ZNF282 |
| FAM102B |
| PPIP5K1 |
| ZACN |
| LOC440173 |
| ZNF517 |
| WDYHV1 |
| ZMIZ2 |
| ZNF250 |
| TAOK2 |
| GBAP1 |
| HDGFL2 |
| SLF2 |
| RPA1 |
| CEP128 |
| EDC3 |
| NPEPPS |
| ZNF620 |
| HIST1H3H |
| ZMIZ1 |
| CENPB |
| ZSCAN5A |
| SNAPC2 |
| DMPK |
| SUGP2 |
| PPP1R37 |
| MEF2D |
| NSD2 |
| CCNJ |
| ENPP4 |
| SMARCD1 |
| LCORL |
| LYSMD1 |
| MBD3 |
| MAP4K4 |
| AMZ2P1 |
| ZNF718 |
| GOLPH3L |
| GAS8 |
| C11orf21 |
| CASC3 |
| TULP4 |
| MDC1 |
| MAP4K2 |
| SPACA9 |
| C1orf35 |
| BRD3 |
| CIC |
| MAPKBP1 |
| NT5C3A |
| MFSD5 |
| BNIPL |
| DNMT3A |
| MOB2 |
| PLIN3 |
| C6orf48 |
| AP2B1 |
| RAP1GAP |
| TAF6 |
| TCF20 |
| IFI27L1 |
| SMARCAL1 |
| SAE1 |
| AP4M1 |
| C1orf229 |
| C6orf163 |
| ERMP1 |
| LRRC37A2 |
| PRDM4 |
| CBX1 |
| CAMTA1 |
| ZNF16 |
| POLD3 |
| CNP |
| RAD51D |
| IQCE |
| HIST1H2BE |
| NUP62 |
| FBXW8 |
| ZNF783 |
| SLC9A6 |
| EIF4ENIF1 |
| INTS3 |
| PHOSPHO2-KLHL23 |
| HPDL |
| TIGD7 |
| CYFIP2 |
| ZNF354A |
| GORAB |
| PLCD4 |
| SUPT5H |
| CNOT3 |
| SOX18 |
| LOC100133315 |
| TCOF1 |
| DVL2 |
| SPPL3 |
| TRIM28 |
| ARHGEF9 |
| TASP1 |
| CD59 |
| USP20 |
| DIP2A |
| NMUR2 |
| ATAT1 |
| CSNK1G1 |
| RFXANK |
| POLR3A |
| OR6T1 |
| STK38L |
| ZNF628 |
| ACLY |
| ZNF271P |
| COLGALT1 |
| TRIM3 |
| PIP4P1 |
| ZNF76 |
| MAU2 |
| SLC37A1 |
| SERF1B |
| GOLGA2P7 |
| KANSL3 |
| CNPPD1 |
| PHF6 |
| ABCC5 |
| RILPL1 |
| PPDPF |
| SPATA2 |
| FAM98C |
| C20orf194 |
| PROCA1 |
| RGS5 |
| RABEP2 |
| MPV17 |
| PTPRA |
| COX19 |
| PLA2G6 |
| PHF13 |
| RUSC1 |
| C6orf136 |
| HDHD5-AS1 |
| NCOA3 |
| PYGO2 |
| HIST3H2BB |
| LINC00299 |
| KIAA1522 |
| TMEM44 |
| ZKSCAN8 |
| SMAD5 |
| RRAS |
| MED16 |
| ZSCAN22 |
| TMEM184B |
| ASH1L |
| GALNT10 |
| CUL7 |
| INAFM1 |
| ACD |
| LINC01119 |
| RNF44 |
| TMEM120B |
| PKD1L3 |
| RFPL3S |
| USP11 |
| RALGAPB |
| RALY |
| ZBTB45 |
| SMYD5 |
| ZBTB9 |
| EP400 |
| TMUB2 |
| RAI1 |
| TBCB |
| KIAA2026 |
| LCOR |
| FOXO4 |
| RIN2 |
| RSU1 |
| ANO8 |
| ARID1A |
| PHIP |
| POGZ |
| CSPG4 |
| INO80E |
| LRRC27 |
| VASH2 |
| INTS2 |
| MRPL42P5 |
| PTOV1 |
| CHRNA2 |
| SCNM1 |
| RBM15B |
| KDM3B |
| VPS11 |
| LASP1 |
| HSPA1L |
| OR51E2 |
| TTBK2 |
| DLGAP4 |
| ABHD17A |
| DEDD2 |
| ZNF212 |
| ECE1 |
| SGSM2 |
| APC |
| MTR |
| CHD4 |
| UBTD2 |
| MED25 |
| ARL6IP6 |
| SENP1 |
| SELENON |
| MED12 |
| WWC3 |
| SNRPA |
| TSC22D4 |
| ILF3 |
| GGA3 |
| ZBTB39 |
| TRRAP |
| ZNF767P |
| TUBD1 |
| LSM7 |
| BTN2A3P |
| PRKRIP1 |
| ZBTB8A |
| AP3B1 |
| CSNK2A1 |
| RFTN1 |
| RPS6KB1 |
| PLRG1 |
| OSBP |
| SP100 |
| TAF13 |
| YY1 |
| SPRR1A |
| NUP54 |
| LTN1 |
| NEUROD2 |
| TRMT5 |
| MICU1 |
| CRBN |
| JAK1 |
| LINC00989 |
| CXorf38 |
| PNPLA4 |
| NLRC4 |
| FBXL5 |
| OXR1 |
| NEK9 |
| ELP3 |
| SCYL1 |
| RTN4 |
| AICDA |
| C6orf120 |
| CPNE9 |
| ABRAXAS1 |
| ANTXRL |
| C7orf71 |
| SNORA5C |
| ARF4 |
| AP5M1 |
| ABHD17B |
| TRAF3 |
| IAH1 |
| PIGY |
| IMPA1 |
| REXO2 |
| TMED10 |
| CCM2 |
| RPS27L |
| WDR44 |
| RAD21L1 |
| AIF1 |
| FBXO25 |
| ANKRD18DP |
| ZDHHC20 |
| C12orf29 |
| USP4 |
| TMC8 |
| MIS12 |
| CAPZA2 |
| FASTKD2 |
| PPA2 |
| NFE2L2 |
| BSDC1 |
| SYNJ2BP |
| VCPIP1 |
| USO1 |
| AIMP1 |
| CT47A11 |
| HLA-DMA |
| PRR32 |
| RAB18 |
| CIPC |
| NUBP1 |
| NFIC |
| CCDC50 |
| RBL2 |
| GFY |
| CMTR2 |
| HEBP1 |
| TTN |
| MRPS22 |
| MIOS |
| SPAG7 |
| LGALS17A |
| MFSD9 |
| ALG5 |
| MKLN1 |
| KDELR2 |
| MLF2 |
| TMEM134 |
| HOXD12 |
| SEL1L |
| AKNA |
| HSD17B12 |
| HCRTR2 |
| OSTF1 |
| NUMB |
| VGLL2 |
| SPAG9 |
| OR2T5 |
| KLHL10 |
| RBM18 |
| TMEM126A |
| NGLY1 |
| UCKL1-AS1 |
| BMP2K |
| ALG11 |
| RAB21 |
| IMP4 |
| EIF2S1 |
| MTIF3 |
| KRT26 |
| KBTBD13 |
| LOC643802 |
| SBDS |
| TRMT10C |
| CNBP |
| RPL36A-HNRNPH2 |
| CLIC4 |
| ANKRD44 |
| LINC01973 |
| ATP5MC3 |
| CRK |
| KPNA3 |
| IQCF2 |
| IFITM4P |
| WBP4 |
| MFSD1 |
| LRRC40 |
| SPN |
| TANK |
| FGFR1OP2 |
| CAPZA1 |
| HERPUD1 |
| DNAJB4 |
| TVP23B |
| PHF8 |
| GCFC2 |
| AGO1 |
| MRPL32 |
| NUS1 |
| PRKCZ |
| TK2 |
| GLOD4 |
| PSMA3 |
| LRRC74B |
| SPTY2D1 |
| TMEM174 |
| MPP5 |
| YIPF4 |
| GPR15 |
| DDX28 |
| HMGB1 |
| IFNGR1 |
| MAP7D3 |
| PPM1A |
| KBTBD7 |
| CDK10 |
| OR10A7 |
| TRUB2 |
| TXNDC11 |
| ATXN3 |
| B2M |
| SBDSP1 |
| NDUFS1 |
| RPL21P44 |
| PPP1R26-AS1 |
| FAM3C |
| KLK2 |
| ODF3L2 |
| ZBTB16 |
| ATP5F1B |
| SELENOI |
| MAP3K5 |
| CMTM6 |
| SEC24A |
| HPRT1 |
| MRPS28 |
| UBE3C |
| CYP11B1 |
| MYBPC1 |
| C1QTNF12 |
| PSCA |
| EFR3A |
| TMEM167A |
| RNFT1 |
| TVP23C |
| IFI27 |
| MTIF2 |
| PPTC7 |
| FAM186B |
| TBATA |
| COG8 |
| PDE12 |
| AKAP14 |
| ITM2B |
| 7-Mar |
| GPR179 |
| OR52A1 |
| CORO7 |
| DISC2 |
| OR5A2 |
| PAICS |
| CSK |
| VKORC1L1 |
| NINJ1 |
| MBIP |
| COQ9 |
| RPL21 |
| FNDC3A |
| CTAGE11P |
| SCYL2 |
| ITSN2 |
| CDHR1 |
| ATL3 |
| EN1 |
| OR5M3 |
| TMEM37 |
| MDM2 |
| PRMT9 |
| TPT1 |
| FOXN3-AS1 |
| ELOA |
| MRPL54 |
| CAB39 |
| SEC24D |
| ECEL1P2 |
| TDRP |
| RWDD2B |
| EPAS1 |
| ABCE1 |
| CTSB |
| TMEM123 |
| PDCD6IPP2 |
| HADHB |
| RAB43 |
| NARS2 |
| TMEM256 |
| C1QBP |
| PPID |
| LOC100132741 |
| ITSN1 |
| DMD |
| KLK14 |
| TBC1D15 |
| C20orf141 |
| ELFN1 |
| ZC3H12C |
| CAMK2D |
| OR2Y1 |
| PRB4 |
| MAP2K4 |
| HIST1H4K |
| PINX1 |
| YARS |
| PRDX6 |
| PRAM1 |
| FAT4 |
| LCN12 |
| GALK2 |
| NDUFV2 |
| SERPINA1 |
| MRPL16 |
| COQ4 |
| ABCC13 |
| MUC7 |
| KLF6 |
| PTK2B |
| TMPRSS13 |
| EHBP1 |
| MMADHC |
| TMPRSS15 |
| SETD3 |
| HIST1H4J |
| IFT88 |
| RBBP8NL |
| CCDC36 |
| CTAGE4 |
| GPR52 |
| CDKL1 |
| ANKRD52 |
| PRKAG2 |
| FAM126A |
| SERINC2 |
| CARMIL2 |
| CEBPD |
| PPP2R5C |
| CNIH1 |
| CCT6B |
| IGFN1 |
| FERMT2 |
| APOL1 |
| AK3 |
| IL13RA1 |
| CABP2 |
| ABCD4 |
| ATG2A |
| LHX9 |
| LYZL2 |
| ALDH9A1 |
| TMEM170B |
| PLPBP |
| BEND4 |
| LINC00963 |
| TPRG1L |
| LPXN |
| GSAP |
| EFCAB1 |
| SERP1 |
| KIAA1551 |
| LINC01588 |
| ANKRD9 |
| EIF5A |
| ATG2B |
| ANO1 |
| CTSL |
| SLC9A3R2 |
| COQ2 |
| PSG6 |
| BZW1 |
| DDX3X |
| FGD4 |
| ARSB |
| SVIL-AS1 |
| PAFAH2 |
| ODF3B |
| PIP4K2A |
| SRP54 |
| GPALPP1 |
| SNORA53 |
| TRIM5 |
| CCDC185 |
| TMEM176B |
| CTAGE15 |
| DEFB104B |
| DYNLL2 |
| TMLHE |
| CYTH4 |
| PGGT1B |
| DLST |
| INTS6 |
| OR2B11 |
| MRM3 |
| PDE1B |
| MBTPS2 |
| AGO4 |
| DENND5B |
| ARHGAP45 |
| USP15 |
| SMIM12 |
| FAM91A1 |
| HECTD3 |
| ID2 |
| OR10A3 |
| DESI1 |
| INSIG2 |
| SNX9 |
| SEC23A |
| BNIP2 |
| CTBS |
| DDX19B |
| IFNAR1 |
| ABCA13 |
| COPZ2 |
| POLD4 |
| HOOK1 |
| API5 |
| PTPN3 |
| MPP1 |
| PPP2CB |
| HCG11 |
| PTGR2 |
| OR6X1 |
| CTRC |
| TBX21 |
| ACKR2 |
| TNF |
| WWC2 |
| CRADD |
| DNAJA1 |
| WWC2-AS2 |
| ARHGAP20 |
| OTOP3 |
| LINC01141 |
| CARNMT1 |
| ACOT13 |
| LINC00244 |
| RDX |
| ZMYND15 |
| PTMS |
| HLA-DPB2 |
| MYH3 |
| MRPL35 |
| ARHGAP15 |
| OR4F6 |
| LARS2 |
| MGST2 |
| SPCS3 |
| RMDN2 |
| C10orf82 |
| C2CD3 |
| SNORA66 |
| EIF3J |
| LYN |
| PRCP |
| CNDP2 |
| PSD4 |
| MOB1A |
| C11orf86 |
| ADGRE1 |
| TFB2M |
| CNST |
| RAPGEF2 |
| HIST1H1A |
| ABCD3 |
| RBKS |
| MIXL1 |
| HLA-DRB1 |
| GTF3A |
| AP1AR |
| FCGRT |
| CRYBB1 |
| UBE2D3 |
| MYLK |
| DUSP16 |
| RAB33B |
| TTLL11 |
| RNF168 |
| SLC30A7 |
| C11orf24 |
| COLEC11 |
| DNAJB9 |
| ARHGAP42 |
| MAX |
| HLA-DMB |
| MTRNR2L6 |
| NBPF6 |
| GFM1 |
| TRPV3 |
| STOM |
| IRF2 |
| ESD |
| GTF3C2-AS1 |
| LSMEM2 |
| A2M |
| DHRS12 |
| GFI1 |
| ECI2 |
| USPL1 |
| MEPE |
| DEFB121 |
| MIA2 |
| TNFRSF10B |
| GLUL |
| HPS5 |
| PAWR |
| SAT2 |
| TACO1 |
| GRHL2 |
| NMD3 |
| RNF6 |
| CTSLP8 |
| GOLGA4 |
| GVINP1 |
| OXSM |
| SDHA |
| MGLL |
| ZNF770 |
| HBQ1 |
| PTP4A1 |
| TRMT10A |
| SHMT2 |
| PEBP1 |
| KLHL8 |
| CTSO |
| TTC39B |
| RAB17 |
| LGALS8 |
| DEFB123 |
| LOC644189 |
| NFKBIA |
| NHLRC2 |
| PEX13 |
| CXCL16 |
| SLC16A5 |
| USP31 |
| RRN3 |
| SAT1 |
| PSMA3-AS1 |
| NR1H3 |
| SLC19A2 |
| SIGIRR |
| MCEE |
| RNF217 |
| SLCO1A2 |
| UTP3 |
| MPO |
| PAQR3 |
| MRPS31 |
| NIPSNAP3A |
| REPS2 |
| ACTR3C |
| PAN2 |
| TRPC5 |
| GBP5 |
| TRAPPC3L |
| NADK |
| CBR4 |
| PANX1 |
| TMEM252 |
| FMR1NB |
| CLIC3 |
| GABARAPL1 |
| PITPNM2 |
| FAM71B |
| REPS1 |
| C2orf78 |
| NUDT12 |
| MATK |
| ACOT4 |
| FBXO31 |
| EPHB1 |
| KLF9 |
| LY96 |
| LINC01354 |
| MAP3K2 |
| OR2M7 |
| RORA |
| GBE1 |
| ECHS1 |
| GCSH |
| IGFBP2 |
| LGALS2 |
| PNPO |
| IL2RG |
| GSPT1 |
| GIMAP4 |
| THNSL1 |
| ARHGAP30 |
| ARHGEF26 |
| SEC24B |
| ZFP36 |
| TAAR3P |
| BTK |
| NKIRAS1 |
| CXCL6 |
| GFRA2 |
| PYHIN1 |
| DEFB107A |
| SMIM19 |
| DUSP2 |
| GOLIM4 |
| FAHD2A |
| MIR99AHG |
| EMP3 |
| MERTK |
| PSG5 |
| TMED5 |
| SETD7 |
| LOC100335030 |
| HLA-DRA |
| LOC100293612 |
| TTPAL |
| ACADVL |
| ACER1 |
| GPR146 |
| SLC2A7 |
| C12orf66 |
| BCL6 |
| NIT2 |
| TRHDE-AS1 |
| LINC00700 |
| GPA33 |
| AKAP3 |
| LGMN |
| SELPLG |
| RALYL |
| SFTA2 |
| SCARNA9L |
| SNORA62 |
| SNORD108 |
| GRPEL1 |
| SLC6A2 |
| GPR174 |
| NRN1 |
| HCST |
| PLEKHH1 |
| TMEM150B |
| SH3D19 |
| FANCC |
| LINC00314 |
| BAZ1A |
| ITGA9 |
| OR5M9 |
| NHLRC3 |
| PARP15 |
| PLSCR4 |
| FASLG |
| C11orf1 |
| HEATR4 |
| KLF8 |
| FAM180B |
| USP30 |
| HLA-DPB1 |
| PTS |
| ERICH1 |
| PTGDR |
| HLA-DOA |
| ZDHHC19 |
| OR10G9 |
| ENPEP |
| HSD17B4 |
| ZC2HC1C |
| JUN |
| FGFBP1 |
| NANOGNB |
| HLA-DQB1 |
| SALL3 |
| INPP1 |
| CARD16 |
| PEX3 |
| NTSR2 |
| KAT2B |
| CD68 |
| MYCT1 |
| TBRG1 |
| OR4S2 |
| LILRB4 |
| LOC100131532 |
| OR2T12 |
| GPHN |
| LOC440700 |
| ID1 |
| GPR87 |
| ADTRP |
| OR4C16 |
| TMBIM6 |
| FSCB |
| CARD17 |
| LILRA1 |
| SPDYC |
| ADRB1 |
| FOXI3 |
| SORL1 |
| HSDL2 |
| ATP8B4 |
| AIFM1 |
| TMEM192 |
| UBE2L6 |
| ABHD5 |
| DUSP1 |
| AHR |
| GNPNAT1 |
| UBL3 |
| ACOX1 |
| OR2AK2 |
| FAM160A1 |
| UGP2 |
| DIPK1C |
| FAM180A |
| NDUFAF1 |
| ADK |
| RARRES3 |
| ZNF680 |
| C16orf78 |
| MAP2K1 |
| FNIP2 |
| OR4C11 |
| KRT74 |
| KRT33B |
| TYROBP |
| DUSP6 |
| NBPF3 |
| MCCC1 |
| PTPN22 |
| ITGB2 |
| ETFRF1 |
| PMPCA |
| TNFRSF10A |
| FAAH |
| METAP1D |
| WARS |
| SOX5 |
| SLC31A1 |
| NPY |
| LOC641746 |
| MLANA |
| MORC1 |
| CDC37L1 |
| MYD88 |
| TBXAS1 |
| CABP4 |
| IKZF1 |
| ADPRM |
| C11orf71 |
| ZNF330 |
| GNE |
| CES3 |
| CLPX |
| RNA18SN5 |
| OR4M1 |
| FTLP10 |
| ACOT1 |
| ZNF619 |
| MAOB |
| L1TD1 |
| EPB42 |
| COBLL1 |
| SLC19A3 |
| SFTPC |
| SYT10 |
| TOR1AIP2 |
| ERO1B |
| NMUR1 |
| DIO3 |
| CEBPB |
| POLE4 |
| FAM49A |
| LOC100506497 |
| ADCYAP1R1 |
| KRTAP26-1 |
| SLC22A11 |
| TNFAIP8L2 |
| CRYZ |
| COG3 |
| C1orf53 |
| WISP3 |
| SMIM14 |
| HIBCH |
| LOC220729 |
| KRT25 |
| FGF4 |
| HADH |
| PIK3R5 |
| IGFLR1 |
| VENTXP7 |
| SDHB |
| ARL5B |
| TP53INP1 |
| OR52W1 |
| SPPL2A |
| SCO1 |
| LARP4 |
| CSF1R |
| F8 |
| LOC729080 |
| HINT2 |
| KRT39 |
| CLPB |
| SPRYD4 |
| LRRC70 |
| ERRFI1 |
| TGFBR3 |
| HLA-DPA1 |
| C16orf87 |
| EDEM1 |
| ERLIN1 |
| SIAE |
| LRCH1 |
| CDNF |
| FXN |
| PGM1 |
| GMPR |
| PRF1 |
| USP38 |
| MPDZ |
| ART5 |
| ACR |
| ACTRT3 |
| FYB2 |
| UAP1 |
| CD86 |
| SHPK |
| ARL11 |
| LOC286177 |
| SIGLEC11 |
| ANTXR2 |
| CLIC2 |
| HCLS1 |
| IL12RB1 |
| PDLIM2 |
| EGR1 |
| RNASE4 |
| PRAMEF14 |
| SLC35F4 |
| SNORA13 |
| GIMAP5 |
| ALB |
| CA3 |
| SLCO2B1 |
| SULT1A2 |
| HRASLS2 |
| SGPP1 |
| PPP1R32 |
| ABCA1 |
| TIGD2 |
| NMRK1 |
| CCDC198 |
| CES1P1 |
| CCR2 |
| HAR1A |
| NRG4 |
| RNF152 |
| GNRH2 |
| RAMP3 |
| ISPD |
| SSX5 |
| STARD5 |
| LILRA6 |
| IDNK |
| ACO1 |
| PUS10 |
| SDHD |
| LDHC |
| CD82 |
| FAM83A |
| OR10G4 |
| PROC |
| GPR171 |
| ST6GAL1 |
| OR9Q2 |
| DKFZp779M0652 |
| FABP4 |
| CASP1 |
| C11orf65 |
| NUDT15 |
| ATF3 |
| CTAGE10P |
| ARMH1 |
| UFM1 |
| MT4 |
| APOH |
| SATB1 |
| ALAD |
| HLA-DRB3 |
| NCR1 |
| NT5E |
| FCRL6 |
| ECHDC2 |
| MYF6 |
| LOC100130880 |
| DECR1 |
| NEFH |
| SUGT1P4-STRA6LP |
| SLC37A4 |
| PITPNM3 |
| TRIM35 |
| FYB1 |
| C1R |
| POLR2M |
| FAM71D |
| GYG2 |
| CECR7 |
| SLC30A1 |
| PPARG |
| KRTAP3-3 |
| SRL |
| PLA2G16 |
| SLC46A2 |
| ARPP21 |
| HCK |
| NOSTRIN |
| HK3 |
| BMP10 |
| CPAMD8 |
| NUDT16 |
| CD4 |
| SLC16A4 |
| FDX1 |
| PLAC8 |
| LACC1 |
| ACAA1 |
| ROS1 |
| GFOD1 |
| LPIN2 |
| GNAT2 |
| CTAGE7P |
| UBXN8 |
| C9orf72 |
| TMEM131L |
| GZMM |
| ADGRG6 |
| FRMD4B |
| RETSAT |
| LINC01465 |
| SYPL2 |
| WNT5B |
| PDLIM5 |
| CFL2 |
| FRMD1 |
| MUT |
| EBPL |
| HMGCL |
| FRMD7 |
| GRHPR |
| KIR3DL2 |
| SPI1 |
| ABRACL |
| CR1L |
| ADA2 |
| TUBA3D |
| SLC37A2 |
| CPT2 |
| SELENOP |
| SNORA55 |
| ZBTB20 |
| TAF7L |
| KLHL2 |
| SLC34A1 |
| HIGD1A |
| CDC14B |
| SLC9B2 |
| SYT1 |
| SLC9A9 |
| HAR1B |
| LST1 |
| SLC25A20 |
| MAGEA8 |
| ARHGAP10 |
| PCBP1-AS1 |
| RSC1A1 |
| NUDT7 |
| EVA1A |
| NCKAP1L |
| PARP4 |
| C5 |
| SLC15A2 |
| C21orf91 |
| IL1R1 |
| ETFA |
| ALPK1 |
| SOWAHC |
| POR |
| MMAA |
| C2orf16 |
| SH2D1A |
| CD14 |
| OSBPL11 |
| CD1D |
| TSPAN11 |
| PROK1 |
| GC |
| MYO1B |
| MPC1 |
| NEAT1 |
| SLC24A4 |
| TMEM269 |
| TRIB1 |
| TEK |
| AQP11 |
| NKG7 |
| PROS1 |
| SNORD115-28 |
| IL36RN |
| OLAH |
| GPR137B |
| NNMT |
| OR10G7 |
| CXorf21 |
| CD247 |
| SLC5A1 |
| FOLH1B |
| PAIP2B |
| MAST4 |
| SLC39A5 |
| PLPP3 |
| CD84 |
| HLF |
| TFF2 |
| KIAA0087 |
| STBD1 |
| ATP7B |
| HSD17B11 |
| PLCB2 |
| MS4A7 |
| TPD52L1 |
| TARP |
| CXCL11 |
| CLEC3B |
| TRAT1 |
| STYK1 |
| FAM170B-AS1 |
| PDE2A |
| CCL24 |
| NXPH1 |
| VAV1 |
| IL10RA |
| CXCR6 |
| IL7R |
| SPATA6L |
| HP |
| NDRG2 |
| KIR2DL5A |
| FNDC4 |
| SOD2 |
| SNORD18C |
| ZNF812P |
| PTPN20 |
| LINC00596 |
| OR52A5 |
| C11orf54 |
| CNKSR3 |
| ABHD18 |
| NSMCE1-DT |
| P2RY2 |
| AQP3 |
| FGG |
| ISY1-RAB43 |
| DOCK2 |
| TFEC |
| GK |
| LOC100506253 |
| TTC38 |
| PLA1A |
| TTC36 |
| KLK4 |
| ICAM3 |
| HIBADH |
| PDE6G |
| UGT2B4 |
| AGL |
| C1QA |
| ASS1 |
| PZP |
| FOLH1 |
| CCDC57 |
| LOC100506368 |
| C1QB |
| GK3P |
| IL2RB |
| MYEOV |
| FXYD1 |
| ZFHX4 |
| SLC52A1 |
| CNGB3 |
| SLC16A11 |
| FGA |
| PTPRC |
| FAS |
| CYP2S1 |
| MYO1F |
| GOLGA8S |
| LCP1 |
| RIDA |
| CA13 |
| DEFB119 |
| PECR |
| GRIK1 |
| LARP1B |
| CPS1-IT1 |
| CCDC71L |
| TNIP3 |
| ROPN1L |
| PLA2G7 |
| SNORD93 |
| BCO1 |
| LOC200772 |
| CYB5A |
| CFH |
| BCL2L10 |
| CAVIN2 |
| TUT7 |
| CD38 |
| CYP4V2 |
| OAF |
| CYP27A1 |
| TDGF1 |
| PVALB |
| TUBE1 |
| IL1RAP |
| KLK15 |
| ACAA2 |
| AQP6 |
| SGK1 |
| MICALCL |
| ACADM |
| TCIM |
| FOLR2 |
| ARMC6 |
| ADGRA3 |
| PEX11A |
| SAMD4A |
| ADAMTS13 |
| LINC01220 |
| CRYL1 |
| MCCC2 |
| SRGN |
| PRB1 |
| TLR4 |
| KLRB1 |
| RASL11A |
| CTCFL |
| TRANK1 |
| SCP2 |
| ALDH2 |
| KRTAP27-1 |
| BIN2 |
| CYP7B1 |
| PTH1R |
| ADGRE4P |
| NAPSA |
| C1QTNF9 |
| F12 |
| C1RL |
| ZMYND12 |
| PDK4 |
| OR5AK4P |
| RHOB |
| MYOT |
| STEAP1B |
| DPEP3 |
| CCDC17 |
| LILRB2 |
| DCAF11 |
| IL18 |
| TLR1 |
| HGF |
| KRTAP4-12 |
| ACAT1 |
| PSD3 |
| RILP |
| NLRP3 |
| OXNAD1 |
| STEAP1 |
| LINC01621 |
| IGSF6 |
| HLA-DQA2 |
| CCRL2 |
| ASGR1 |
| ZFAND5 |
| KLRK1 |
| SLC6A13 |
| IL33 |
| ALAS1 |
| IVD |
| MPPED1 |
| SLC25A30 |
| C4A |
| GAMT |
| TMEM92 |
| SERPINA10 |
| PMEL |
| LOC100505622 |
| KCNJ8 |
| PHYH |
| RSPH10B |
| ADI1 |
| SERPINB8 |
| GBP1 |
| TNFSF11 |
| ADRA1B |
| SNORD18B |
| DDI2 |
| CTPS1 |
| NSUN6 |
| SLC14A1 |
| CLEC9A |
| CPEB3 |
| LNX2 |
| HEMGN |
| FOXO1 |
| SYTL5 |
| CD302 |
| CYP11A1 |
| PITPNM2-AS1 |
| TMEM45A |
| NAPRT |
| ETFDH |
| GPR183 |
| SLC16A2 |
| NXPE1 |
| LINC00308 |
| LONP2 |
| SLC4A4 |
| TMEM26 |
| HLA-DQA1 |
| CYP2B6 |
| ZNF648 |
| DAPP1 |
| SYT9 |
| ADRA2B |
| HLA-DRB4 |
| LIN7A |
| TNFRSF10D |
| LONRF3 |
| IL18R1 |
| USP12 |
| HMOX1 |
| ANGPTL6 |
| ITPR2 |
| NTN4 |
| GCH1 |
| PCCB |
| MYOM2 |
| SNX10 |
| KRT27 |
| PER3 |
| ACACB |
| C10orf105 |
| PCSK6 |
| C5AR2 |
| OSTN |
| NGF |
| MSR1 |
| TCP10L |
| AZGP1P1 |
| DCXR |
| FGR |
| CTSLP2 |
| SLC31A2 |
| SOCS2 |
| CYR61 |
| SYCE1 |
| C1S |
| TSKU |
| CXCL12 |
| OPLAH |
| MYRIP |
| FCGR3A |
| RGN |
| GPR65 |
| LINC01010 |
| LILRB3 |
| SNORD115-32 |
| UGT2B11 |
| KIR2DS4 |
| FCER1G |
| RPGRIP1 |
| ACPP |
| DEFA4 |
| CD274 |
| MNDA |
| TEC |
| CFHR3 |
| CSAD |
| CDH23 |
| CD300C |
| SYBU |
| GCDH |
| PXMP2 |
| HNMT |
| CLDN2 |
| DOK2 |
| SNORA28 |
| SLC16A10 |
| GADD45B |
| CD53 |
| ALAS2 |
| EGR3 |
| STX11 |
| C19orf38 |
| LINC00885 |
| UGT2B10 |
| MEFV |
| AKR7A2P1 |
| SLC10A5 |
| SRD5A1 |
| NUDT6 |
| C2orf88 |
| IKBKG |
| TNFRSF11B |
| USP30-AS1 |
| PTCRA |
| CHN2 |
| RCL1 |
| FCN1 |
| SH2D1B |
| HLA-DRB6 |
| SLC39A14 |
| GBP1P1 |
| ADIRF |
| CYBB |
| SFXN2 |
| PRSS8 |
| NTF3 |
| KIR2DL2 |
| VWA8 |
| OVCH1 |
| ABCC9 |
| SLC38A2 |
| CADM2 |
| MGC27382 |
| BCKDHB |
| KLF4 |
| NAMPT |
| HS3ST3B1 |
| HSPB9 |
| SNORD115-23 |
| PTGS2 |
| MT1A |
| LRRC25 |
| CTSW |
| MAN1C1 |
| MTHFD1 |
| DRD1 |
| LILRA3 |
| LINC00632 |
| CST7 |
| FGL1 |
| FLVCR2 |
| FKBP5 |
| CD300A |
| EVI2B |
| CFB |
| MEGF10 |
| MT2A |
| LURAP1L |
| AKR7A3 |
| MSMO1 |
| SELENBP1 |
| SLC16A7 |
| HGD |
| MT1L |
| SERPING1 |
| FGF10 |
| GABRP |
| LINC00612 |
| ABCA6 |
| NKX3-1 |
| LINC02085 |
| NAV2-AS4 |
| FGL2 |
| DPF3 |
| ABHD6 |
| SEC14L2 |
| ANG |
| NPL |
| MOV10L1 |
| PLEK |
| IFNG |
| DPEP2 |
| ACSL5 |
| ECHDC3 |
| SPATA41 |
| C1orf162 |
| SNORD115-7 |
| ATP11C |
| SLC27A2 |
| ACSL1 |
| LY6E |
| RCAN1 |
| CFD |
| CD80 |
| CLEC7A |
| RIPOR2 |
| INSIG1 |
| AP2A1 |
| LRRC2 |
| TMIGD3 |
| CYTIP |
| A1BG |
| CALHM6 |
| FABP3 |
| PHYHD1 |
| SIGLEC7 |
| MGC12916 |
| MIR4290HG |
| PRH2 |
| HSD11B1 |
| RBMY2EP |
| TM6SF2 |
| CAPN3 |
| RAG1 |
| CD72 |
| ESR1 |
| SERPINA7 |
| ACSS3 |
| RNF125 |
| GADD45G |
| AHSG |
| ENTPD5 |
| SARDH |
| USH2A |
| C1QC |
| SNORA14B |
| SLA |
| AMHR2 |
| RND3 |
| DHODH |
| MST1 |
| GSTZ1 |
| CPED1 |
| ACADS |
| ANGPTL1 |
| MPEG1 |
| PLIN1 |
| SLC35D1 |
| VSIG4 |
| SNORA14A |
| ACY1 |
| APOC3 |
| CXorf66 |
| ACAD11 |
| SERPINA11 |
| AKR7L |
| PCOLCE2 |
| LILRB1 |
| ALDH1A1 |
| ST7-AS2 |
| LRRTM1 |
| CDA |
| PI16 |
| CFI |
| PC |
| GPR182 |
| F2 |
| CTSS |
| SIGLEC1 |
| ST3GAL6 |
| GPLD1 |
| CCR1 |
| LILRA4 |
| VMO1 |
| TMEM56 |
| N4BP2L1 |
| SGMS2 |
| SNORD115-1 |
| NUDT16P1 |
| PTGR1 |
| ACOX2 |
| KIR2DS2 |
| IPCEF1 |
| FITM1 |
| PALM3 |
| DNASE1L2 |
| PLEK2 |
| MAJIN |
| UGT2B7 |
| GLTPD2 |
| FPR1 |
| NLN |
| ASB4 |
| MS4A6A |
| AOAH |
| KLRC1 |
| CRYAA |
| LINC00525 |
| NOL4 |
| PRKCB |
| SIGLEC9 |
| HBB |
| MT1B |
| CD200R1 |
| CD36 |
| HHIP-AS1 |
| HORMAD2 |
| MGST1 |
| CD244 |
| FAM13A |
| NFAM1 |
| C14orf180 |
| AASS |
| ADCY10 |
| PALM2 |
| PDCD1LG2 |
| ADRA1A |
| IGF1 |
| LINC00605 |
| ACADSB |
| TRPV6 |
| EXOC3L4 |
| FLT3 |
| SNORD115-3 |
| CARMIL3 |
| ADRB2 |
| GNA14 |
| HAVCR2 |
| DEPDC7 |
| EOMES |
| CPVL |
| DNASE2B |
| IGFALS |
| KNG1 |
| CRYBG1 |
| CCR3 |
| FAM74A1 |
| DHRS2 |
| LILRA5 |
| NXF3 |
| TENT4B |
| RGPD1 |
| SNORD115-4 |
| PMP2 |
| KIR2DL4 |
| PHACTR2-AS1 |
| ACOT6 |
| CD180 |
| HBA2 |
| CCL14 |
| CHL1 |
| IL1RN |
| KYNU |
| NLRP11 |
| HAAO |
| SLC27A5 |
| PBLD |
| PROKR2 |
| SCARNA9 |
| LYPD2 |
| RBP5 |
| S100A9 |
| ALDH6A1 |
| BTNL8 |
| CD163 |
| TMPRSS2 |
| DSG2-AS1 |
| GJB3 |
| GLYCTK |
| SLC25A15 |
| CBS |
| SAMSN1 |
| KBTBD11 |
| TCTEX1D1 |
| DHRS9 |
| ITIH3 |
| C6 |
| CNGA1 |
| SCIMP |
| FMO3 |
| PTPRO |
| AKR1C6P |
| ASPA |
| STEAP4 |
| LILRB5 |
| FBP1 |
| TLR2 |
| CYP2J2 |
| FREM2 |
| FTCD |
| RGS18 |
| LINC00939 |
| CIDEB |
| RTL4 |
| SNORD115-5 |
| LIFR |
| CR1 |
| PLIN4 |
| KCND3 |
| GPT2 |
| RFPL4A |
| IL1RL1 |
| LOC157273 |
| ACE2 |
| KDM8 |
| MT1E |
| SLITRK6 |
| AKR1C8P |
| ADHFE1 |
| AZGP1 |
| NADK2 |
| NUGGC |
| LY6E-DT |
| RETREG1 |
| ADGRE3 |
| OR52N2 |
| MOCOS |
| CA5A |
| HBD |
| AGTR1 |
| MIR3945HG |
| IGFBP1 |
| SELE |
| MFSD2A |
| LINC00421 |
| SLC38A4 |
| EHHADH-AS1 |
| TLR8 |
| MDGA2 |
| VNN1 |
| AMDHD1 |
| TFPI2 |
| CYP4A22 |
| MST1P2 |
| RDH5 |
| FOS |
| ITIH4 |
| FOXP2 |
| ITIH1 |
| PCK2 |
| PTGER2 |
| ITGAD |
| GLOD5 |
| MIP |
| SLCO4A1 |
| GREM2 |
| LCN2 |
| BCO2 |
| HRG |
| KLRF1 |
| MT1X |
| ADH1A |
| STEAP3 |
| COL6A6 |
| FCAMR |
| FCGR2C |
| HPD |
| PLGLB1 |
| DIO3OS |
| ALDOB |
| MOGAT1 |
| PRODH2 |
| LPAL2 |
| AOX1 |
| CXCL2 |
| MASP1 |
| LILRA2 |
| MRO |
| VXN |
| C4BPA |
| AGXT |
| CHAD |
| TDO2 |
| CLTRN |
| P2RY13 |
| GSTA2 |
| MFAP3L |
| EHHADH |
| ACSM3 |
| AADAT |
| ALDH8A1 |
| COL25A1 |
| CYP4F3 |
| AQP7 |
| FAM74A3 |
| CLEC4GP1 |
| LINC01018 |
| GSTA5 |
| LCAT |
| GSTA3 |
| KLKB1 |
| BCHE |
| PCK1 |
| S100A8 |
| FOSB |
| ACSM2B |
| GPM6A |
| CD160 |
| ADH1C |
| ZBTB10 |
| CCDC196 |
| GHR |
| ASPDH |
| FPR2 |
| SAA3P |
| NOTUM |
| CMTM2 |
| IL10 |
| LINC01348 |
| CCBE1 |
| KLRD1 |
| PPBP |
| UGT3A1 |
| SLC46A3 |
| CCL4 |
| CLRN3 |
| PLG |
| SLC17A2 |
| LRCOL1 |
| KCNN2 |
| LINC01625 |
| PSAT1 |
| HMGCS2 |
| MUM1L1 |
| AFM |
| NBPF7 |
| GLS2 |
| AKR1D1 |
| SAA4 |
| ABCB4 |
| TCP10 |
| ACSM5 |
| UGT1A6 |
| SERPINA4 |
| CES1 |
| AKR1C4 |
| CYP4F12 |
| HSD17B6 |
| DNASE1L3 |
| LPA |
| GPT |
| UPB1 |
| SUCNR1 |
| C8A |
| BAAT |
| SERPINC1 |
| UGT1A8 |
| F11 |
| LYVE1 |
| CTH |
| OTC |
| SLC2A2 |
| KMO |
| FETUB |
| TPPP2 |
| GBA3 |
| SPP2 |
| HHIP |
| P2RY12 |
| TTPA |
| AADACP1 |
| VIPR1 |
| GFRA1 |
| FCGR2B |
| CETP |
| CFHR1 |
| GMNC |
| TREH |
| F9 |
| SLC6A12 |
| RSPO3 |
| SLC22A10 |
| TAT |
| MOGAT2 |
| MT1F |
| CFP |
| GNMT |
| ADH6 |
| CYP2C9 |
| CYP1A1 |
| ACY3 |
| TERB2 |
| ANKRD55 |
| CYP4F2 |
| MT1M |
| CLC |
| ACOT12 |
| SLCO1B1 |
| CD209 |
| SLC22A3 |
| CES5AP1 |
| ANXA10 |
| RDH16 |
| MBL2 |
| GDA |
| CYP2C19 |
| CYP4F11 |
| CFHR4 |
| CYP2C8 |
| PRG4 |
| SDS |
| BOK-AS1 |
| FCAR |
| GJB2 |
| DSG1 |
| NDST3 |
| ARG1 |
| SLC25A18 |
| ANGPTL3 |
| MASP2 |
| LDHD |
| CFHR2 |
| GLYATL1 |
| ASPG |
| CFTR |
| S100A12 |
| SHBG |
| MT1IP |
| FAM83A-AS1 |
| HAO1 |
| SPIC |
| GSTA7P |
| DEFA3 |
| FAM151A |
| UROC1 |
| TIMD4 |
| CXCR1 |
| SULT2A1 |
| FAM9B |
| F13B |
| ADH4 |
| LINC01554 |
| SLC51A |
| CES1P2 |
| ITLN1 |
| OIT3 |
| APOC4 |
| KCNJ10 |
| CYP4A11 |
| NAT2 |
| APOA5 |
| CES5A |
| HAL |
| COLEC10 |
| HAMP |
| BHMT |
| SULT1B1 |
| C9 |
| PGA3 |
| DAO |
| SLC10A1 |
| MT1H |
| BMPER |
| HEPN1 |
| C3P1 |
| CYP2A13 |
| ALDH1L1 |
| CYP8B1 |
| SULT1E1 |
| CXCR2P1 |
| IDO2 |
| SLC22A1 |
| SAA1 |
| FCN3 |
| CLEC12A |
| HSD17B13 |
| DNMT3L |
| HGFAC |
| GLYAT |
| MTTP |
| GCGR |
| GBP7 |
| ETNPPL |
| APOF |
| DBH |
| AQP9 |
| TMEM82 |
| AVPR1A |
| UGT2A2 |
| THRSP |
| CPN2 |
| HAO2 |
| ADH1B |
| MT1DP |
| SLC25A47 |
| MME |
| ACADL |
| PLA2G2A |
| CYP3A4 |
| CYP39A1 |
| HJV |
| HEPACAM |
| PON1 |
| NR1I2 |
| CYP1A2 |
| SRD5A2 |
| FAM99B |
| CYP2E1 |
| ABCB11 |
| ADGRG7 |
| CLEC4M |
| GYS2 |
| MT1G |
| FAM99A |
| CCL23 |
| CLEC4G |
| SAA2 |
| MARCO |
| LINC01093 |
| CRHBP |
| CNDP1 |
| CYP3A43 |
| STAB2 |
| SLCO1B3 |
| UPP2 |
| CD5L |
| CFHR5 |
| FCN2 |
| CLEC1B |
