# Supplementary material for: Gene Expression Characteristics of Liver Tissue Reveal the Underlying Pathogenesis of Hepatocellular Carcinoma
Source: Biomed Res Int. 2021 Oct 4;2021:9458328. doi: 10.1155/2021/9458328 (PMC8506137; doi:10.1155/2021/9458328)
Supplement: Supplementary 2 — Table S2.36231 Functional enrichment results. [file 9458328.f2.docx]

| Table S2. 36231 functional enrichment results | | |
| --- | --- | --- |
| GO_MF | GO_CC | GO_BP |
| 5569 | 3281 | 27381 |

附表2
