# Supplementary material for: Gene Expression Characteristics of Liver Tissue Reveal the Underlying Pathogenesis of Hepatocellular Carcinoma
Source: Biomed Res Int. 2021 Oct 4;2021:9458328. doi: 10.1155/2021/9458328 (PMC8506137; doi:10.1155/2021/9458328)
Supplement: Supplementary 3 — Table S3.356 ncRNA-Module target pairs. [file 9458328.f3.docx]

| Table S3.356 ncRNA-Module target pairs | |
| --- | --- |
| module | pivot |
| m1 | miR-7703 |
| m1 | miR-6849-3p |
| m1 | FENDRR |
| m1 | miR-98-5p |
| m1 | miR-122-5p |
| m1 | miR-128-3p |
| m1 | MALAT1 |
| m1 | miR-24-3p |
| m1 | miR-503-5p |
| m1 | miR-16-5p |
| m1 | ANCR |
| m1 | let-7e-5p |
| m1 | let-7d-5p |
| m1 | let-7a-5p |
| m1 | miR-4500 |
| m1 | miR-591 |
| m1 | miR-106a-5p |
| m1 | miR-1-3p |
| m1 | miR-340-5p |
| m1 | miR-429 |
| m1 | miR-4465 |
| m1 | miR-27b-3p |
| m1 | miR-373-3p |
| m1 | miR-200c-3p |
| m1 | miR-519d-3p |
| m1 | miR-214-3p |
| m1 | miR-425-5p |
| m1 | miR-758-3p |
| m1 | TUG1 |
| m1 | miR-548s |
| m1 | miR-4435 |
| m1 | miR-3664-5p |
| m1 | miR-30d-5p |
| m1 | miR-30a-5p |
| m1 | miR-30b-5p |
| m1 | let-7i-5p |
| m1 | miR-335-5p |
| m1 | miR-374b-5p |
| m1 | miR-30e-5p |
| m1 | let-7f-5p |
| m1 | miR-27a-3p |
| m1 | miR-202-3p |
| m1 | miR-136-5p |
| m1 | miR-21-5p |
| m1 | let-7c-5p |
| m1 | miR-491-5p |
| m1 | miR-147a |
| m1 | miR-374a-5p |
| m1 | miR-135b-5p |
| m1 | miR-29a-3p |
| m1 | miR-129-5p |
| m1 | miR-873-5p |
| m1 | miR-363-3p |
| m1 | miR-383-5p |
| m1 | miR-4459 |
| m1 | miR-4433a-3p |
| m1 | miR-4793-3p |
| m1 | miR-6720-5p |
| m1 | miR-155-5p |
| m1 | miR-101-3p |
| m1 | miR-5580-3p |
| m1 | miR-193b-3p |
| m1 | miR-1277-5p |
| m1 | miR-1224-3p |
| m1 | miR-137 |
| m1 | miR-548as-5p |
| m1 | miR-548b-5p |
| m1 | miR-323b-5p |
| m1 | miR-559 |
| m1 | miR-29b-3p |
| m1 | miR-6778-5p |
| m1 | miR-361-5p |
| m1 | miR-574-5p |
| m1 | miR-193a-3p |
| m1 | miR-532-3p |
| m1 | miR-1260a |
| m1 | miR-548e-3p |
| m1 | miR-450a-1-3p |
| m1 | miR-6745 |
| m1 | miR-363-5p |
| m1 | miR-548a-3p |
| m1 | miR-5582-3p |
| m1 | miR-1178-3p |
| m1 | miR-6867-5p |
| m1 | miR-3152-5p |
| m1 | miR-3177-5p |
| m1 | miR-449a |
| m1 | miR-6499-3p |
| m1 | miR-508-5p |
| m1 | miR-7110-3p |
| m1 | miR-190b |
| m1 | miR-6791-3p |
| m1 | miR-520a-5p |
| m1 | miR-3919 |
| m1 | miR-2467-3p |
| m1 | miR-133b |
| m1 | miR-452-3p |
| m1 | miR-4695-5p |
| m1 | miR-6749-3p |
| m1 | miR-7114-5p |
| m1 | miR-5047 |
| m1 | let-7b-3p |
| m1 | miR-671-5p |
| m1 | miR-222-3p |
| m1 | miR-525-5p |
| m1 | miR-4680-5p |
| m1 | miR-142-5p |
| m1 | miR-6838-5p |
| m1 | miR-1185-1-3p |
| m1 | miR-4643 |
| m1 | miR-4306 |
| m1 | miR-6817-3p |
| m1 | miR-1227-3p |
| m1 | miR-3652 |
| m1 | miR-4739 |
| m1 | miR-5192 |
| m1 | miR-6812-3p |
| m1 | miR-1343-3p |
| m1 | miR-6852-5p |
| m1 | miR-1206 |
| m1 | miR-663a |
| m1 | miR-1908-5p |
| m1 | miR-6830-3p |
| m1 | miR-4652-3p |
| m1 | miR-6878-5p |
| m1 | miR-302b-5p |
| m1 | miR-1238-3p |
| m1 | miR-3611 |
| m1 | miR-216a-3p |
| m1 | miR-362-5p |
| m1 | miR-3140-3p |
| m1 | miR-6795-3p |
| m1 | miR-3909 |
| m1 | miR-3146 |
| m1 | miR-24-1 |
| m1 | miR-4657 |
| m1 | miR-616 |
| m1 | DMGDH |
| m1 | SSR4P1 |
| m1 | miR-6753-3p |
| m1 | miR-3128 |
| m1 | miR-139-3p |
| m1 | miR-6753-5p |
| m1 | miR-4802-3p |
| m1 | miR-8072 |
| m1 | miR-219a-1-3p |
| m1 | miR-6715b-5p |
| m1 | miR-630 |
| m1 | miR-4782-5p |
| m1 | miR-3651 |
| m1 | miR-4800 |
| m1 | miR-4435-2 |
| m1 | SNORA19 |
| m1 | FLJ36777 |
| m1 | STARD13-IT1 |
| m1 | LOC646903 |
| m1 | miR-7162-5p |
| m1 | miR-490-5p |
| m1 | miR-7152-3p |
| m1 | miR-641 |
| m1 | miR-4762 |
| m1 | miR-4677 |
| m1 | LINC01617 |
| m1 | SNORD78 |
| m1 | LOC400958 |
| m1 | LOC100133985 |
| m1 | miR-5002-3p |
| m1 | NPCDR1 |
| m1 | LINC00221 |
| m1 | miR-3940-5p |
| m2 | MALAT1 |
| m2 | miR-514a-3p |
| m2 | miR-130a-3p |
| m2 | miR-17-5p |
| m2 | miR-193b-3p |
| m2 | miR-378a-3p |
| m2 | miR-221-3p |
| m2 | miR-107 |
| m2 | miR-29c-3p |
| m2 | miR-365a-3p |
| m2 | miR-30d-5p |
| m2 | miR-30a-5p |
| m2 | miR-374a-5p |
| m2 | miR-30b-5p |
| m2 | miR-26b-5p |
| m2 | miR-382-5p |
| m2 | miR-192-5p |
| m2 | TUG1 |
| m2 | miR-186-5p |
| m2 | let-7b-5p |
| m2 | miR-494-3p |
| m2 | let-7e-5p |
| m2 | miR-539-5p |
| m2 | miR-106b-5p |
| m2 | miR-499a-5p |
| m2 | miR-20b-5p |
| m2 | miR-182-5p |
| m2 | miR-590-3p |
| m2 | miR-146b-5p |
| m2 | miR-93-5p |
| m2 | miR-302e |
| m2 | miR-520b |
| m2 | miR-1297 |
| m2 | miR-125a-3p |
| m2 | miR-320c |
| m2 | miR-215-5p |
| m2 | miR-371a-5p |
| m2 | miR-372-5p |
| m2 | miR-4514 |
| m2 | miR-374b-5p |
| m2 | miR-302c-3p |
| m2 | miR-520c-3p |
| m2 | let-7d-5p |
| m2 | miR-208a-3p |
| m2 | miR-520e |
| m2 | miR-495-3p |
| m2 | miR-381-3p |
| m2 | miR-190b |
| m2 | miR-921 |
| m2 | miR-6816-3p |
| m2 | miR-4649-5p |
| m2 | miR-6729-5p |
| m2 | miR-106a-5p |
| m2 | miR-508-5p |
| m2 | miR-885-3p |
| m2 | miR-596 |
| m2 | miR-5096 |
| m2 | miR-548az-5p |
| m2 | miR-3174 |
| m2 | miR-4528 |
| m2 | miR-30c-5p |
| m2 | miR-320a |
| m2 | miR-136-5p |
| m2 | miR-5581-5p |
| m2 | miR-33b-5p |
| m2 | miR-26a-5p |
| m2 | miR-4714-3p |
| m2 | let-7f-5p |
| m2 | let-7c-5p |
| m2 | miR-373-3p |
| m2 | miR-217 |
| m2 | miR-548i |
| m2 | miR-3163 |
| m2 | miR-548ak |
| m2 | miR-548ay-5p |
| m2 | miR-302d-3p |
| m2 | miR-6829-3p |
| m2 | miR-4495 |
| m2 | HULC |
| m2 | miR-3675-3p |
| m2 | miR-371b-5p |
| m2 | miR-4698 |
| m2 | miR-8088 |
| m2 | miR-6793-5p |
| m2 | miR-1912 |
| m2 | miR-4507 |
| m2 | miR-4634 |
| m2 | HELLPAR |
| m3 | miR-381-3p |
| m3 | miR-3666 |
| m3 | miR-18a-5p |
| m3 | miR-30b-5p |
| m3 | miR-432-5p |
| m3 | miR-25-3p |
| m3 | miR-223-3p |
| m3 | miR-4500 |
| m3 | miR-1229-3p |
| m3 | miR-6734-5p |
| m3 | miR-26b-5p |
| m3 | miR-92a-3p |
| m3 | TUG1 |
| m3 | miR-494-3p |
| m3 | miR-590-3p |
| m3 | miR-4755-5p |
| m3 | miR-124-3p |
| m3 | miR-299-3p |
| m3 | miR-338-3p |
| m3 | FENDRR |
| m3 | miR-155-5p |
| m3 | miR-186-5p |
| m3 | let-7b-5p |
| m3 | miR-103a-3p |
| m3 | miR-1275 |
| m3 | miR-33b-5p |
| m3 | miR-135a-5p |
| m3 | miR-448 |
| m3 | miR-181b-5p |
| m3 | miR-375 |
| m3 | miR-504-5p |
| m3 | miR-548bb-3p |
| m3 | miR-548ac |
| m3 | miR-548z |
| m3 | miR-135b-5p |
| m3 | miR-127-3p |
| m3 | miR-548d-3p |
| m3 | miR-17-3p |
| m3 | miR-3922-3p |
| m3 | miR-4723-5p |
| m3 | miR-6130 |
| m3 | miR-3074-5p |
| m3 | miR-6747-3p |
| m3 | miR-423-5p |
| m3 | miR-6752-5p |
| m3 | miR-6834-5p |
| m3 | miR-378j |
| m4 | miR-6875-3p |
| m4 | miR-6508-5p |
| m4 | miR-150-5p |
| m4 | miR-4635 |
| m4 | miR-7851-3p |
| m4 | miR-3116 |
| m4 | miR-3171 |
| m5 | miR-4695-3p |
| m5 | miR-134-3p |
| m5 | miR-708-5p |
| m5 | miR-4768-3p |
| m5 | miR-6828-5p |
| m5 | miR-4459 |
| m5 | miR-7641 |
| m5 | miR-6832-5p |
| m5 | miR-2392 |
| m5 | miR-3689f |
| m6 | miR-2114-5p |
| m6 | miR-335-5p |
| m6 | miR-4747-3p |
| m6 | miR-20b-3p |
| m6 | miR-2861 |
| m6 | miR-4441 |
| m6 | miR-4289 |
| m6 | miR-4327 |
| m6 | miR-887-3p |
| m7 | miR-185-5p |
| m7 | miR-5088-5p |
| m7 | miR-1-3p |
| m7 | miR-6828-3p |
| m7 | miR-5006-5p |
| m7 | miR-423-3p |
| m7 | miR-370-3p |
| m7 | miR-4276 |
| m7 | miR-6738-3p |
| m7 | miR-6780b-3p |
| m7 | miR-629-3p |
| m7 | miR-623 |
| m8 | miR-548m |
| m9 | miR-6504-3p |
| m9 | miR-7157-5p |
| m9 | miR-6814-5p |
| m9 | miR-2113 |
| m10 | miR-1248 |
| m10 | miR-3157-5p |
| m10 | miR-4691-3p |
| m10 | miR-548a-3p |
| m10 | miR-548ar-3p |
| m10 | miR-1306-5p |
| m10 | miR-548az-3p |
| m10 | miR-7153-5p |
