# Supplementary material for: Gene Expression Characteristics of Liver Tissue Reveal the Underlying Pathogenesis of Hepatocellular Carcinoma
Source: Biomed Res Int. 2021 Oct 4;2021:9458328. doi: 10.1155/2021/9458328 (PMC8506137; doi:10.1155/2021/9458328)
Supplement: Supplementary 4 — Table S4.52 TF-Module control pairs. [file 9458328.f4.docx]

| Table S4.52 TF-Module control pairs | |
| --- | --- |
| module | pivot |
| m1 | NFKB1 |
| m1 | STAT3 |
| m1 | RELA |
| m1 | SP1 |
| m1 | SP3 |
| m1 | SREBF1 |
| m1 | PPARA |
| m1 | STAT1 |
| m1 | STAT6 |
| m1 | HNF1A |
| m1 | CEBPA |
| m1 | PPARGC1A |
| m1 | ATF2 |
| m1 | JUN |
| m1 | MEF2D |
| m1 | SPI1 |
| m1 | NFE2L2 |
| m1 | TRERF1 |
| m1 | CIITA |
| m1 | RFX1 |
| m1 | RFX5 |
| m1 | NR1I2 |
| m1 | GLI1 |
| m1 | AES |
| m1 | GLI2 |
| m1 | POU2F2 |
| m1 | RFXANK |
| m1 | RFXAP |
| m1 | ILF3 |
| m2 | E2F1 |
| m2 | BRCA1 |
| m2 | E2F4 |
| m2 | EP300 |
| m2 | KLF5 |
| m2 | TCF7L2 |
| m2 | TP53 |
| m2 | ZNF143 |
| m2 | MYC |
| m2 | ZHX2 |
| m2 | FOXM1 |
| m2 | TBP |
| m2 | DACH1 |
| m2 | HSF2 |
| m3 | CDX2 |
| m3 | AR |
| m3 | TWIST1 |
| m3 | MAZ |
| m4 | REST |
| m6 | HDAC3 |
| m8 | MITF |
| m8 | TFEB |
| m10 | STAT6 |
